# In vivo tractography of human locus coeruleus—relation to 7T resting state fMRI, psychological measures and single subject validity

**DOI:** 10.1038/s41380-022-01761-x

**Published:** 2022-09-18

**Authors:** Thomas Liebe, Jörn Kaufmann, Dorothea Hämmerer, Matthew Betts, Martin Walter

**Affiliations:** 1 Department of Psychiatry and Psychotherapy, University of Jena, D-07743 Jena, Germany; 2 Department of Radiology, University of Jena, D-07743 Jena, Germany; 3 Clinical Affective Neuroimaging Laboratory (CANLAB), D-39120 Magdeburg, Germany; 4 Leibniz Institute for Neurobiology, D-39118 Magdeburg, Germany; 5 Department of Neurology, University of Magdeburg, D-39120 Magdeburg, Germany; 6 Department of Psychology, University of Innsbruck, A-6020 Innsbruck, Austria; 7 Institute of Cognitive Neuroscience, University College London, London, UK-WC1E 6BT UK; 8 Institute of Cognitive Neurology and Dementia Research, Otto-von-Guericke-University Magdeburg, D-39120 Magdeburg, Germany; 9 CBBS Center for Behavioral Brain Sciences, D-39120 Magdeburg, Germany; 10 German Center for Neurodegenerative Diseases (DZNE), D-39120 Magdeburg, Germany; 11 Department of Psychiatry and Psychotherapy, University Tuebingen, D-72076 Tuebingen, Germany; 12 Center for Intervention and Research on adaptive and maladaptive brain Circuits underlying mental health (C-I-R-C), D-07743 Jena, Germany; 13 German Center for Mental Health (DZPG), Site Jena-Magdeburg-Halle, D-07743 Jena, Germany

**Keywords:** Diagnostic markers, Neuroscience

## Abstract

The locus coeruleus (LC) in the brainstem as the main regulator of brain noradrenaline gains increasing attention because of its involvement in neurologic and psychiatric diseases and its relevance in general to brain function. In this study, we created a structural connectome of the LC nerve fibers based on in vivo MRI tractography to gain an understanding into LC connectivity and its impact on LC-related psychological measures. We combined our structural results with ultra-high field resting-state functional MRI to learn about the relationship between in vivo LC structural and functional connections. Importantly, we reveal that LC brain fibers are strongly associated with psychological measures of anxiety and alertness indicating that LC-noradrenergic connectivity may have an important role on brain function. Lastly, since we analyzed all our data in subject-specific space, we point out the potential of structural LC connectivity to reveal individual characteristics of LC-noradrenergic function on the single-subject level.

## Introduction

The locus coeruleus (LC) as the primary source of noradrenaline (NA) in the human brain has been functionally and anatomically studied for decades using histological techniques and electrophysiological measures. Its central importance for key cognitive processes such as cognitive control, arousal and attention, memory, and emotions have widely been confirmed based on task studies in both animal and human experiments [1–4]. Evidence is accumulating for its prominent role in prevalent degenerative brain disorders such as Alzheimer’s [5–8] and Parkinson’s disease [9]. Recent studies have further confirmed involvement of the LC in depression and anxiety disorder [10, 11]. To date, different attempts have been made to gain insight into the functionality and microstructure of the human LC. The anatomical region itself can be depicted in MRI by applying neuromelanin-sensitive sequences [12, 13] to generate measures of LC structural integrity (for reviews see [14, 15]) which have been shown to correlate with proposed psychological capacities of the LC and the mentioned diseases [16–18]. Recently, functional MRI studies have assessed the general interplay between the LC with other brain regions [19–22], disturbances in LC functional connections and structural integrity in aging [23, 24] and associations with antidepressant medication [25]. Recently, we underpinned the former used methods by using neuromelanin-sensitive sequence to extract fMRI signal from the individual subject-specific location of the LC [22].

But one important step in assessing the individual structure of the LC is still missing. Since the functionality of the LC and consequently the disturbances in the associated diseases lie in the spillover of NA throughout its long-reaching nerve fibers [26, 27], the structural connections of the LC and how they relate to individual differences in functional connectivity should be investigated. In the following study, we map the structural fiber connections of the LC to all its important target regions in the human brain using magnetic resonance imaging (MRI). Here, we examine the relationship between structural (23 healthy participants) and functional (18 healthy participants of the same group) LC connectivity in the brain and emphasize the advantages of structural mapping compared to functional connectivity. Thereby, we will show that the structural connectivity is highly related to key psychological trait measures of LC related brain function: alertness and anxiety [28].

Based on previous knowledge from animal studies we hypothesized to uncover LC fibers with widespread connections to the whole neocortex, basal forebrain, limbic system, the thalamus, hypothalamus, and brainstem regions [2], as well as the the thalamus which receives extensive noradrenergic innervation [29]. The anterior cingulate cortex may have sparse LC innervation compared to the posterior cingulate cortex [30] and regarding the neocortical innervation, we expected a dominance of the somatosensory cortex and sparsity in posterior parietal and visual regions [31]. To appropriately measure the exact location of the LC target signal, we aimed to delineate the LC of each participant using neuromelanin-sensitive MRI as performed in our former MRI study [22]. Starting at the individual LC origin, we wanted to trace the LC structural connectivity into the brain using diffusion-weighted magnetic resonance imaging (dMRI). For reduction of reconstruction biases and improvement of biological plausibility of the structural connectome, we aimed to apply the recently developed algorithm of spherical-deconvolution informed filtering of tractograms (SIFT), to reveal the underlying connectivity patterns of the LC in vivo [32]. To learn about the association of structural connections and the functional integration of the LC within other brain regions, ultra-high field 7T resting-state functional MRI (rs-fMRI) data of the subjects were collected with the intention to assess the relationship between the LC blood-oxygen-level-dependent (BOLD) signal and the same brain regions we investigated for structural connectivity. All functional data were processed subject-specific within the segmented anatomical data without using any registration templates. We expected concordance between structural and functional connectivity, at least in highly connected brain areas. To gain insight into practical applicability and into typical LC trait properties, we set out to evaluate how psychological measures are associated with LC structural and functional connectivity. We assessed the State-Trait Anxiety Inventory (STAI, [33]) to relate LC structural connectivity to anxiety [34] and conducted the attention network task (ANT) [35] with a focus on its alertness properties, which are tightly linked to LC noradrenergic control [36]. Based on previous research linking LC signal intensity and corresponding LC cell loss in Alzheimer’s and Parkinson’s to disease vulnerability [37–39], we proposed low fiber count to be a corresponding risk factor for adverse alertness and anxiety scores [27]. Recently, the LC structural connectivity to selected target areas was inspected [40–42], and we aim to extend the ongoing research with our anatomical based tractography method, SIFT2 tractography to get closest to biologically accurate fiber count, whole brain analysis and comparison of our results with 7T rs-fMRI and behavioral data.

## Results

### Characterization and comparison of LC structural connectome on a single subject level

**Fig. 1:**
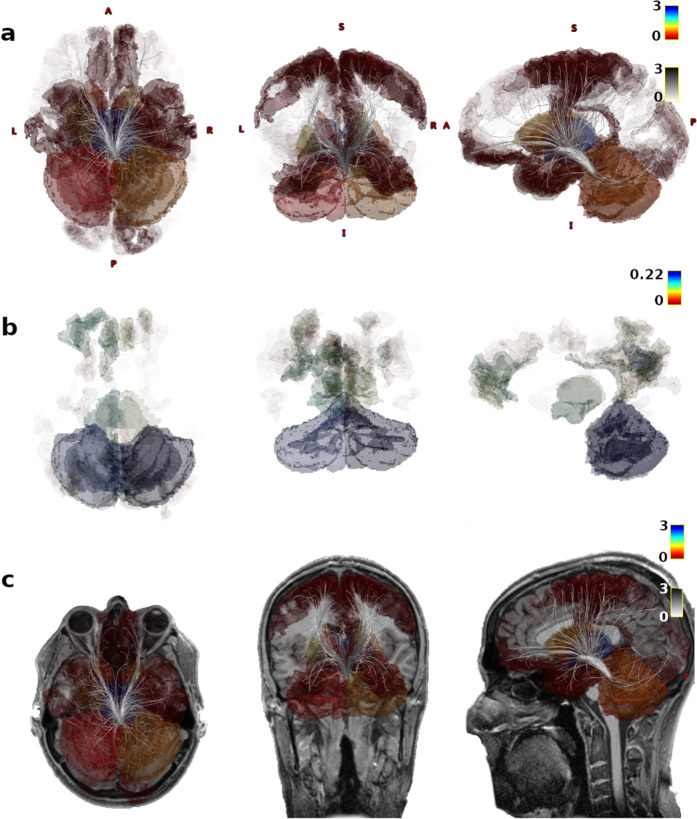
Structural and functional locus coeruleus (LC) connectivity. Illustration of structural (**a**, **c**; SIFT2 filtered tractography) and functional (**b**, 35 min resting-state fMRI) LC connectivity in one example participant. The segmented brain regions represent the individual subjects’ brain anatomy of the areas that are connected with the LC. The opacity and color of those regions illustrate the strength of connectivity where blue colored regions show highest connectivity.

Based on the 3T MRI diffusion data, we computed tractography starting from the individual segmented LC location (identified using a neuromelanin-sensitive 3T TSE sequence) to the whole brain for all subjects separately, with all segmented and anatomically labelled areas of each individual whole brain as possible tractography target regions (based on the Glasser atlas parcellation, see methods section for the whole procedure). Visually we found a very high concordance between structural connections of the LC in vivo with known LC projections determined from prior anatomical knowledge. All subjects showed higher structural connections from the LC to the thalamus, ventral diencephalon, basal ganglia, cerebellum, presubiculum, entorhinal cortex, hippocampus, amygdala, and the nucleus accumbens. Regarding neocortical innervations, a dominance of the basal forebrain, polar frontal cortex, primary motor- and sensory cortex, and mid-to posterior cingulate was evident. The similarity in structural connectivity between the subjects was remarkably high (G-coefficient 0.99, D-study result for a single subject 0.87, see Supplementary Figs. 1 and 2 for an illustration of all subjects). We additionally investigated the functional connectivity of the LC by extracting the blood oxygen level dependent (BOLD) signal in the individually segmented LC area using 7T resting-state MRI. Then, we correlated the timecourse of the haemodynamic LC response to the BOLD signal extracted from all other individually segmented brain areas (see methods section). Figure 1 shows the structural and functional LC connectome of one example participant. Supplementary Figs. 1 and 2 illustrate the raw and z-transformed connectivity values of the structural LC connectome and the corresponding functional connectivity measures of all subjects to provide a direct comparison on a single subject level. The individual results from all subjects demonstrate much higher intersubject variability for functional (G-coefficient 0.80, D-study result for a single subject 0.18) compared to structural connectivity (G-coefficient 0.99, D-study result for a single subject 0.87, Supplementary Fig. 3). Interestingly, subcortical areas such as thalamus and ventral diencephalon, which showed the highest structural connectivity were also highly functionally connected to the LC (Supplementary Fig. 2, first and second rows).

### Group statistics for LC structural connectome and relationship to functional connectivity

**Fig. 2:**
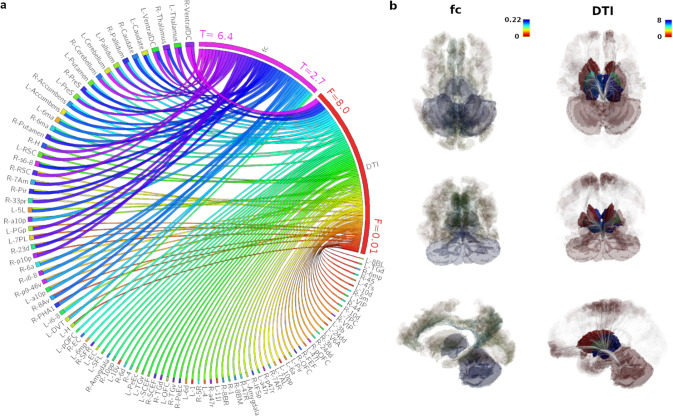
Group statistics and relationship between functional and structural locus coeruleus (LC) connectivity. Regions with high structural connectivity (**a**, DTI) are also strongly functionally (**a**, fc) connected to the LC (FDR *p* < 0.05), most evident in the subcortical thalamic, ventral diencephalic, cerebellar regions and the presubiculum (regions counter-clockwise ordered by strongest structural and functional connections, see also Supplementary Table 3 for Glasser abbreviations). In (**b**), colors and opacity illustrate group statistics of strength of functional (left column, group level FDR *p* < 0.05, *h*-values) and structural connectivity (DTI, right column, group level FDR *p* < 0.05, *F* values) to the whole brain, with blue colored regions representing strongest connectivity.

**Fig. 3:**
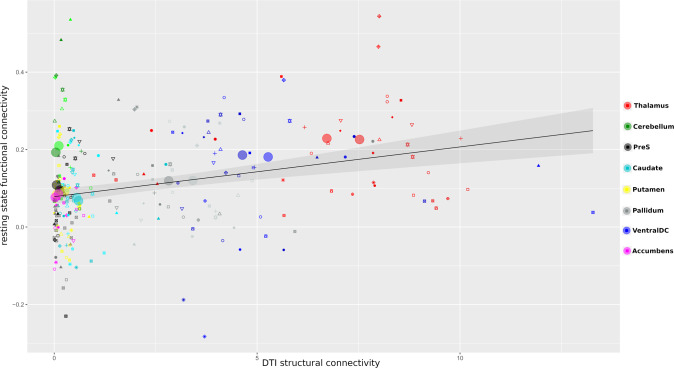
Structural and functional locus coeruleus (LC) whole-brain connectivity showed a linear relationship (Pearson’s *r* = 0.687, *p* = 0.001). Big circles represent mean connectivity values to each brain region (right and left hemisphere) whilst small symbols illustrate connectivity on the individual subject level (e.g., two small red triangles represent LC connectivity to the right and left thalamus for one subject). This association is driven by regions with both high structural and functional connectivity, which reveals that brain areas with high structural connectivity to the LC are also tightly functionally linked to the LC in the resting state, e.g., the thalamus or ventral diencephalon.

Using a one-sample *t*-test, the structural connectivity analyses revealed 249 from 387 areas significantly connected to the LC on the group level (FDR < 0.05). Not surprisingly, given the similarity of the results even evident on a single subject level (Supplementary Figs. 1 and 2), highest *F*-values were found between LC and thalamus, ventral diencephalon, basal ganglia, supplementary motor cortex (6ma subregion), nucleus accumbens, orbitofrontal cortex and medial temporal cortex (presubiculum, entorhinal cortex, hippocampus; Fig. 2, Supplementary Table 1). With respect to LC functional connectivity, highest effect sizes (*h*-values) were found to thalamus, cerebellum, ventral diencephalon, hippocampus, and areas of the posterior cingulate cortex (CONN, FDR < 0.05; Figure 2, Supplementary Table 1). Hereby, we found that both the variability of the functional connectivity and the structural connectivity between LC and certain brain regions were dependent on the connectivity strength, with highly connected brain areas showing lower coefficients of variation than loosely connected regions (Supplementary Fig. 5, Spearman correlation *p* < 0.001). Figure 2 presents the group statistics of the structural and functional LC connectome and Fig. 3 the relationship between the two measures. Brain regions with high structural connectivity to the LC also exhibited strong functional connectivity, although we did not expect that all regions that are anatomically linked to the LC would necessarily also show high corresponding activity in the resting state. Statistically we found a positive linear relationship between fiber count and functional connectivity in brain regions with high structural connectivity (Figure 3, *p* = 0.001), whereas brain regions with sparse LC connectivity did not show such a relationship (Fig. 3). The overall G-coefficient, involving all data, between structural connectivity and functional connectivity demonstrated moderate conformity (G = 0.59). Supplementary Fig. 3 summarizes all possible G-Coefficients for fMRI and DTI results and their interaction with respect to the number of subjects included in the model. For example, in fMRI 14 subjects have to be included into the model to reach an overall good G-coefficient of G > 0.75, whereas in our LC DTI measurements, a single subject already reaches a coefficient of G = 0.87.

### Relationship between LC structural connections and psychological measures

**Fig. 4:**
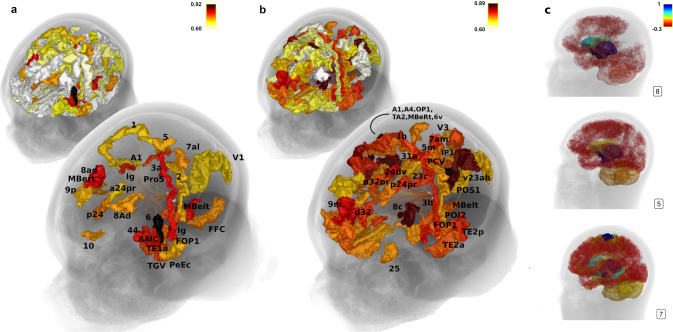
Relationship between locus coeruleus (LC) structural connectivity and psychological traits. Most brain areas (**a**, **b**) that are structurally connected to the locus coeruleus (LC) showed a negative correlation to anxiety (STAI, **a**) and positive correlation to alertness (**b**) scores. All areas depicted in the further-back anatomy illustrations in (**a**, **b**) significantly correlated to anxiety (**a**) and alertness (**b**) with respect to LC structural connectivity, whereas the foreground illustrations single out the correlations with a very high r-coefficient only (*r* > 0.7, see Supplementary Table 3 for Glasser abbreviations). **c** Shows LC whole brain structural connectivity (raw *z*-values, see also Supplementary Fig. 2 for all subjects) of three example subjects: subject ‘8’, whose behavioral score was high in anxiety/low in alertness and who exhibits overall low structural LC connectivity, subject ‘5’ with medium values, and subject ‘7’ with low anxiety/high alertness effects and overall dense LC structural connectivity. **c** Illustrates, how structural connectivity of single subjects may provide a first impression of psychological traits.

To investigate whether LC structural connections are related to psychological measures, all subjects conducted the subjective STAI questionnaire and the alertness properties of the ANT. Both scores showed significant correlations with LC structural connectivity to most brain regions. As proposed, we found an inverse relationship between both measures with lower structural connectivity predicting higher anxiety scores in the STAI and lower scores in the reduction of reaction times with respect to alertness in the ANT. In other words, subjects that showed reduced connections from LC to most of the brain areas tended to exhibit increased anxiety and were not able to improve in a task known to recruit the noradrenergic system (STAI: 161 regions *p* < 0.05 FDR corrected; ANT: 129 regions *p* < 0.05 FDR corrected, Supplementary Table 2). Figure 4b and c show the widespread relevant brain regions for state anxiety and alertness regarding LC structural connectivity, with correlation coefficients coded by color highlighting the areas with the highest correlation *R*-values. Areas with the most prominent relationship to alertness (r coefficients in brackets, FDR corrected *p* < 0.05 for all areas) comprised posterior cingulate areas (parts of Brodmann areas 23 (0.90) and 31 (0.88), POS1 (0.85), 24dv (0.80), PCV (0.78)), auditory (early, core, and secondary auditory areas with right-sided dominance(A1 0.87, A4 0.88, TA2 0.83, MBeRt 0.83), an inferior region of the dorsolateral prefrontal cortex (8c (0.86)), somatosensory association hand and foot area IP1 (0.86) and OP1 (0.85) [43], premotor cortex areas (6v (0.83), FOP1 (0.78), 43 (0.78)), medial prefrontal cortex (9m (0.80)) and posterior insula (PoI1 (0.77)), primary somatosensory regions (3b (0.77), 5m (0.77)), ventral diencephalon (0.58) and left amygdala (0.59) (Figure 4b, Supplementary Table 2 for all statistics, Supplementary Fig. 6). Regarding anxiety, the overall pattern was similar to alertness, however there was more involvement of memory-related regions in the medial temporal cortex (hippocampus (0.56) and perientorhinal/periectorhinal complex (0.49, 0.97)), the insula granular (Ig (0.80)), and the anterior cingulate (p24 (0.74), a24pr (0.71); Figure 4a displays most characteristic regions, Supplementary Fig. 4 delineates group differences between alertness and anxiety *r*-values, Supplementary Table 2 for all statistics). Contrary to the strong structural connectivity findings, we did not find a significant relationship between STAI or alertness scores with functional connectivity (no brain area survived multiple correction, FDR *p* < 0.05).

## Discussion

We describe a procedure how recently developed MRI methods can be applied in vivo to uncover the structural connections of the LC to the whole human brain. Our results are compatible with anatomical and conceptual evidence of the known effective and functional connections of the LC. We compare the structural connections of this key brain region with its functional connectivity using a single-subject approach, revealing higher intersubject-similarity compared to functional connectivity assessments. We found that the brain areas showing highest structural connectivity with the LC also showed the highest functional connectivity. To show a practical use-case of LC structural connectivity, we reveal that key psychological measures related to the LC strongly correlate to the degree of LC connectivity particularly to neocortical brain areas even in healthy young adults. Most importantly, we provide a first view of how psychological inferences on a single-subject level may be carried out using in vivo MRI.

We found connectivity between the LC and most of the cortical and subcortical brain areas, with pronounced connectivity to the thalamus, ventral diencephalon, basal ganglia, motor cortex, cerebellum, amygdala, nucleus accumbens, and temporal brain areas including the entorhinal cortex, presubiculum, and hippocampus. These areas also exhibited strong positive functional connectivity to the LC, which may in part reflect the direct noradrenergic influence of the LC on its target regions: The LC-thalamic structural and functional connectivity is relevant for the weighting of the sensory information, that is brought through bottom-up connections from the whole body to the brain and is relayed within the thalamus [44–46]. The ventral diencephalon includes the hypothalamus, mammillary body, subthalamic nuclei, substantia nigra, red nucleus, lateral geniculate nucleus, and medial geniculate nucleus. Especially the hypothalamus is known to be involved in LC circuits, with the preoptic area regulating wakefulness [47], and important afferents to the LC originate from that [48] area. The noradrenergic innervation of the cerebellum is strikingly relevant for the adaptive ability to coordinate movement [49]. Of great interest is the structural connectivity to the hippocampal and parahippocampal areas comprising the hippocampus, entorhinal cortex, and presubiculum, since those regions are the key areas for memory and they degenerate in Alzheimer’s disease [50–52]. The neocortical LC innervation is attributed to its arousal and wakefulness-promoting action [53], with a dominant role in the basal forebrain [54], and modulatory roles in the motor [55] and sensory cortices [56]. A clear difference regarding LC resting-state functional and structural connectivity was found in the dense fiber projections to the primary somatomotor cortex and no relevant corresponding functional connectivity, whereas equivalently structural and functional connectivity to secondary somatosensory- and motor areas was observed. This is not surprising, since the resting state does not require noradrenergic support of actual movements [57], which are induced in the primary motor cortex, but may integrate sequences of higher-order movements coded in the secondary motor areas in the internal rumination of thoughts [58]. Equally, tactile events, which may trigger primary sensory noradrenergic support, are not present at rest. The both strong functional and structural LC-primary visual area (V1) connectivity can be explained by the dominant involvement of the V1 region in human imagination [59]. In concordance with anatomical findings, the LC structural connectivity to the neocortex was dominated by fibers to sensory-motor regions, and those patterns including the subcortical innervation and are in keeping with known myelinization in neonates, which begins in the same regions [60]. This may reflect the anthropological importance of LC innervation in regions responsible for functions pertaining to basic survival. To potentially relate our results to diagnostics with the aim to uncover psychological traits or disease vulnerability, the method should be stable with respect to a healthy study population. The intersubject-similarity of the structural connections of the LC was remarkably high, especially much higher in comparison to functional connectivity, even though we used to the best of our knowledge the most recent technical possibilities to assess resting-state functional connectivity: long scan time, ultra-high field 7T MRI, visualization of the target structure, single-subject assessment without smoothing and no registration to a template which may result in lost signal. However, we interpret the variability in the functional connectivity as a challenge to learn more about the individual configuration of LC resting-state connections, since the presented patterns may present real differences in state brain connectivity. For potential future group studies, we provide a graph with G-coefficients dependent on the number of included subjects for LC DTI and fMRI measures to give an impression of baseline result variability (Supplementary Fig. 3). Based on our results, LC resting-state fMRI variability starts to decrease and consistent patterns begin to emerge if a minimum of 14 subjects are included and statistically concatenated in the analysis (considering our long fMRI scan time: 35min fMRI run)—whereas in terms of our LC tractography method, even the single subject alone was sufficient to make inferences about structural interconnections within the brain (22 min DTI run).

The similarity of structural connectivity in our subjects per se are promising results, and to begin to understand LC-mediated behavior we demonstrate that LC connectivity correlates with psychological diversity even in healthy young adults. Both alertness and anxiety were strongly associated with LC fiber count to numerous brain regions, in particular frontal control, parietal attention related and paralimbic/limbic regions. The general patterns of alertness and anxiety-related LC structural connectivity were similar—as denoted in the following paragraph, but they differed in sensory processing related regions, which showed especially strong LC innervation related to alertness (Fig. 4b), whereas memory-interoception associated regions exhibited prominent LC innervation related to anxiety (Fig. 4a): Both anxiety and alertness require preparation of fight and flight (in our results BA 6, including frontal eye field [61], frontal cortex “top-down” control of actions and thoughts (we found BA 11, 10, 9m related to alertness [62]; BA 11 and 10 to anxiety [63], parietal regions for stimulus selection [64], the amygdala in emotional attention [65, 66] and the insula region in terms of interoceptive awareness and maintaining alertness (alertness PoI, anxiety Ig, [67–69]. However, alertness depends more on the mapping of actual external sensory signals of the environment (in our results primary sensory cortex area 3; auditory sensory areas, primary visual and visual processing areas [70–72], whereas anxiety relies to a large extent on the retrieval and integration of introspective information (fear circuit involving anterior and posterior cingulate, hippocampus, amygdala [73]. From an information-flow perspective, the outlined patterns of alertness and anxiety-related LC structural connectivity can also be related to the concepts of frontoparietal attention, dorsal attention, and cingulo-opercular (salience) networks [69, 74, 75]. In all those target areas, LC-controlled NA spillover works as a promotor of relevant information and the LC considerably moderates those external and internal directed processes and the permanent integration of their outcome [44, 64, 71, 76, 77]. The relationship of LC fiber count to alertness and anxiety-related regions found here highlights the specificity of structural LC connectivity for LC-related brain functions and its importance for their proper functionality.

Due to the strong association between LC tractography and LC-related psychological measures, we can clearly show the applicability and feasibility of the method compared to functional connectivity and provide a first reasonable view of patterns regarding alertness and anxiety-related cortical innervation. Most strikingly, when focusing on the subjects at the lower and upper end of anxiety and alertness scores, their individual psychological ability to cope with LC and NA related challenges may be evident by looking at the specific patterns of sparsity or density of neocortical LC innervation (Fig. 4c).

Using LC tractography, we can replicate anatomically and functionally known connectivity patterns in vivo and elucidate that LC structural connectivity patterns are related to typical LC trait scores. Importantly, our structural results reach out beyond the indirect nature of functional connectivity, which can be mediated by a third brain part, to a more direct representation of the underlying LC anatomical connections, and thus may allow to uncover the relationship to psychological traits associated with LC function. The clinical applicability of the procedure presented here should be evaluated by comparable studies including clinical populations with patients suffering from psychiatric disorders or neurodegenerative disease. Longitudinal studies might shed light on the processes how LC structural connectivity predicts dynamics in psychiatric symptoms, such as its relation to heightened anxiety or reduced alertness, and potentially aid with response prediction to treatment based on structural connectivity. Especially, individuals with less structural LC connectivity and thus more affected NA system might be more responsive to certain drug therapies compared to individuals with higher connectivity. Gained knowledge about the structural alterations of LC connectivity could then support a more fine-grade diagnostic classification of the patients based on their individual disease pathology.

### Limitations

Since we analyzed a small number of healthy, young, male subjects of western population (Supplementary Table S4), the results demonstrating a relationship between LC structural connectivity and psychological traits are of limited generalizability, but show the general capacity of the method to uncover psychological characteristics even in the individual subject. Importantly, our G-study results indicate, that even a single tractography measurement has sufficient validity to shed light on the individual structural connectivity of the LC.

We found no association of LC rs-fc to ANT and STAI, which could be related to the known low to moderate test-retest reliability of fc [78]. This interpretation is underpinned by the low D-study fc result of a single subject in our findings, which could stem from both MR signal noise components and physiological variability in the LC network. A recent paper suggests that stable fc networks can be generated with 22 subjects in 7T MRI [79]. Our number of 18 included subjects is somewhat lower, but it is based on a scan time twice as long, which should result in an increase in stability. However, at the same time it should be noted, that even single subject studies are able to identify relevant patterns of BOLD fc if the protocols used are suitable in duration in 7T MRI [80], suggesting that many of the features established in our study can be expected to replicate in a bigger study sample.

It would be desirable in future studies to delineate the LC brainstem nucleus using automated methods to overcome potential variability in manual segmentation as conducted here. Nevertheless, in respect to the voxel size of the DTI and fMRI sequences applied in this study, small variability in human delineation of the nucleus is neglectable, since the fine-grade delineated LC masks are transformed to the DTI and fMRI data and the signal extraction is limited to the one to two millimeter-resolution of the main sequences.

In terms of registration of anatomical, diffusion and functional MRI data, extensive visual inspection was performed to guarantee exact alignment of the scans. Our 3T tractography data may have especially benefited from the advanced top-up-distortion correction, whereas for the functional 7T MRI data, the widely used fieldmap-based distortion correction was applied.

## Conclusion

Localization and subject space-based structural connectivity of the LC is a promising method to gain information about noradrenergic-related brain functionality on a single-subject level. The high intersubject-stability, its concordance with functional connectivity and associations with psychological measures emphasize and encourage its applicability in clinical trials.

## Methods

### Participants

Twenty-five male subjects (mean age 24.8 ± 4.2) were recruited by public advertisement. For inclusion and exclusion criteria we refer to the Supplementary Methods. The study was approved by the institutional ethical review board of the University of Magdeburg, and all subjects gave written informed consent in accordance with the Declaration of Helsinki. The collected data of the study are available on request from the authors.

### Behavioral assessments

STAI [33] scores (see Supplementary Methods) were assessed before 7T functional MRI scan one and two and before scan three (see section ‘MRI data acquisition’). Both scores showed a high internal consistency and test-retest reliability (Cronbach’s alpha = 0.88, intraclass correlation coefficient = 0.785) and we calculated the mean of the two measurements to create a stable baseline for further analyses. The ANT [81] can be described as a modified version of the Erikson flanker task [82] disentangling human attention networks (see Supplementary Methods).

### MRI data acquisition

Structural image acquisition and the acquisition of a neuromelanin-sensitive sequence was performed using a Siemens MAGNETOM Prisma 3 T MRI scanner with syngo MR E11 software and a 64-channel head coil as published previously [22] (Supplementary Methods).

Diffusion weighted images were acquired with a monopolar single-shot spin echo EPI sequence on the same scanner with the following parameters: TE = 74 ms; TR = 4970 ms; flip angle *α* = 90°; parallel GRAPPA acceleration factor = 2, matrix: 130 × 130; FOV = 208 × 208 mm^2^; spatial resolution = 1.6 × 1.6 × 1.6 mm^3^; multiband acceleration factor = 2; phase-encoding direction: anterior > > posterior; 228 isotropically distributed diffusion sensitization directions (38 at b = 1.000 s/mm^2^, 76 at b = 2.000 s/mm^2^, and 114 at b = 3.000 s/mm^2^) and 14 b = 0 s/mm^2^ images (interspersed throughout the acquisition) were collected. The sampling scheme was designed according to Caruyer (http://www.emmanuelcaruyer.com/q-space-sampling.php; [83]. To generate appropriate fieldmaps to correct for susceptibility-induced distortions, nine b = 0 s/mm^2^ images with reversed phase encoding (posterior > > anterior) were also acquired. The total scan duration was 22 min 31 s.

Functional MRI data were collected using a Siemens MAGNETOM 7 T MRI scanner with Siemens Syngo VB17 software and a 32-channel head coil using a multi-band accelerated T2*-weighted echo-planar imaging (EPI) sequence. We acquired eyes-closed resting-state fMRI data with total scan duration of 35 min. For parameters of the 7T fMRI sequence we refer to the Supplementary Methods.

### Definition of individual LC location

The LC was manually segmented in the subjects by a radiologist experienced in neuroradiology as described in our previous study [22] (Supplementary Methods).

### Brain parcellation

T1 images were processed using the FreeSurfer pipeline [84] version 7.0 with default parameters which included defining ROIs according to the HCP MMP 1.0 atlas [85]. The output of every subject was checked visually by viewing the subcortical segmentation and the white and pial surfaces using the freeview tool available in FreeSurfer and two subjects were excluded due to insufficient anatomical parcellation. The resulting segmentations (aparc- and aseg-files) comprised all cortical and subcortical regions (180 atlas regions on each cortical hemisphere, plus 19 subcortical regions (2 × 9 plus brainstem)). In total 379 nodes were defined. Using the MRtrix [86] tools mrcalc and mrtransform the brainstem region was excluded and replaced by the LC region (Supplementary Fig. 7 for an illustration of parcellation).

### dMRI preprocessing

DICOM to NIFTI conversion was carried out using dcm2niix (https://github.com/rordenlab/dcm2niix). Afterward, the data were denoised with the dwidenoise tool and Gibbs ringing artifacts were removed with mrdegibbs tool of MRtrix. The FMRIB software library (FSL, University of Oxford, https://fsl.fmrib.ox.ac.uk/fsl/) version 6.0.1 was used for the correction of EPI distortions, eddy current artifacts, and subject movements along with the dMRI scans. All corrections were simultaneously done using the topup/eddy procedure [87]. To improve the brain mask estimation using the dwi2mask tool a bias field correction of the data was done before applying the dwi biascorrect tool and the -ants option [88]. Using dwi2respose (with the “dhollander” algorithm for multi-shell data [89]), we estimated response functions from the preprocessed diffusion-weighted data. These were then used to estimate fiber orientation distribution (FOD) based on 8th order constrained spherical deconvolution using dwi2fod [90]. Specifically, the msmt_csd algorithm [91], which facilitates the computations of three separate FODs for gray-matter (WM), white-matter (GM), and cerebrospinal fluid (CSF) based on multishell data was used. A global intensity normalization of the FODs was done (MRtrix command mtnormalise) to get comparable FODs between subjects.

### Fiber tracking and connectome construction

Anatomical images and parcellations were affine transformed to the undistorted dMRI space using the FSL tool flirt as a rigid body transformation [92]. To take advantage of anatomical constrained tractography (ACT; [93]) the tool 5ttgen implemented in MRtrix was applied to the T1 images based on masks for the GM/WM-boundary which were created using the MRtrix tool 5tt2gmwmi. Probabilistic tractography was performed in two ways: (a) whole-brain probabilistic tractography by randomly seeding 50 million fibers within the GM/WM mask and (b) seed-based tractography with the binary LC mask as seed and 20 million fibers for each subject. The MRtrix tool tckgen with default parameters assisted by ACT, the -backtrack option and a maximal path length of 200 mm was used. After the fiber tract generation both tracts were combined for each subject using tckedit and the spherical-deconvolution Informed Filtering of Tractograms (SIFT2) procedure (MRtrix command tcksift2) was applied [32]. Finally, the SIFT2 outputted streamlines were parcellated into a set of 379 regions of the HCP MMP 1.0 atlas (MRtrix command tck2connectome) [32]. For visualization with mrview streamlines were extracted from the tractograms based on their assignment to parcellated nodes (MRtrix command connectome2tck).

### fMRI preprocessing

The fMRI preprocessing pipeline was similar to our previous publication [22], but no registration of the subjects into MNI space and no smoothing of the data was performed (Supplementary Methods). Five subjects had to be discarded because of strong EPI distortion and consecutive insufficient registration to the anatomical template.

### Statistical analysis

For statistical analysis, SPSS Version 20 and R Version 4.0 [94] were used. For intraclass correlation calculation, Generalizability Theory [95] was applied ([96] for an review of the Generalizability Theory method, Supplementary Methods).

For calculation of a possible linear relationship of functional and structural connectivity on the group level, we computed Pearson permutation correlation coefficients in R with 50,000 iterations, with brain region-specific averaged z-transformed *h*-values extracted from CONN served as functional connectivity measure and brain region-specific averaged z-transformed SIFT2 fiber count served as the structural measure. We split this investigation to examine regions with high and low fiber count (z > 0, z < 0, with 0 representing the mean of the data) after revealing a significant positive relationship of all data (*p* < 0.001). For calculation of individual regions’ LC connectivity variability, we calculated the coefficient of variation (the ratio of the standard deviation to the mean) of positively functionally connected regions (z > 0) and for structurally connected regions with high fiber count (z > 0). The coefficients of variation of the single regions were then Spearman-correlated to the mean connectivity strength of the respective regions using SPSS (Supplementary Fig. 5). Group statistics of functional connectivity data were performed by CONN built-in functions. For ROI-to-ROI functional connectivity analysis, a matrix of Fisher-transformed bivariate correlation coefficients between the individual LC masks’ time series and individual Freesurfer segmented brain regions was individually calculated. Then, a ROI-to-ROI analysis was performed, with NBS based [97] intensity thresholding (computing the sum of test statistic values across all connections comprising the component as an alternative to ’extend’ thresholding, which simply counts the number of connections, equivalent to ‘cluster mass’ as known in cluster-based statistics [98] and seed-level threshold to an FDR *p* < 0.05 after permutation testing was performed (1000 iterations) to account for the total number of connections included in the analysis, as suggested by default CONN settings. T-statistic values and h values, as a measure of effect size, were reported, with *h* values representing the mean Fischer transformed pairwise correlations between LC and the connected ROI’s. CONN calculation formulas are presented in the supplementary material. Group statistics of structural connectivity data were performed with network-based statistics (NBS, [97]) built-in FDR function performing a one-sample test based on 100,000 permutations. The relationship of STAI and ANT alertness scores and LC structural connectivity was assessed with one-sided Pearson permutation correlations with 50,000 iterations in R. Only brain areas with structural connectivity in at least 10 subjects were included in the analysis [99]. The results were FDR corrected for multiple comparisons using R. STAI and ANT scores, and STAI and ANT r-correlation coefficients, that were then used to distinguish the strength of the relationship of STAI and ANT scores with LC structural connectivity to certain brain regions (Fig. 4), did not correlate with each other (Spearman correlation *p* = 0.212 and *p* = 0.394, respectively).

### Data illustrations

Figures 1, 2b and 4 were created with the mrview tool of Mrtrix [86] (Supplementary Methods). Figure 2a was created with Circos Table Viewer [100]. The data panels in Fig. 3 were created with R [94].

## Supplementary information

Supplementary Material
