# Supplementary material for: In vivo tractography of human locus coeruleus—relation to 7T resting state fMRI, psychological measures and single subject validity
Source: Mol Psychiatry. 2022 Sep 18;27(12):4984–93. doi: 10.1038/s41380-022-01761-x (PMC9763100; doi:10.1038/s41380-022-01761-x)
Supplement: Supplementary file 1 — Supplementary Material [file 41380_2022_1761_MOESM1_ESM.pdf]

Supporting Information Text

Supplementary methods

Participants. Our inclusion criteria comprised the absence of a current medical condition and history of major psychiatric illness as determined by medical history, physical examination, blood laboratory tests, electrocardiography, and toxicology findings. Previously, subjects who met the following criteria have been excluded: Subjects with a history of drug or alcohol dependency or abuse within the proceeding 6 months and subjects with serious unstable illness including respiratory, cardiovascular (including ischemic heart disease), endocrinologic, hepatic, renal, gastroenterology, neurologic, immunologic, or hematologic disease, subjects with uncorrected hypothyroidism or hyperthyroidism and subjects with one or more seizures without a clear and resolved etiology. We also excluded subjects with any illness likely to alter brain morphology and/or physiology like diabetes or uncontrolled hypertension and if any metallic (ferromagnetic) implants (heart pacemaker, aneurysm clips) or tattoos were present. The subjects were required to be off current medication for at least 6 weeks prior to the inclusion.

Behavioural assessments - STAI. We assessed the German X1 (trait) version of the STAI questionnaire, comprising the 20 statements "I feel calm", "I feel secure", "I am tense", "I feel strained", "I feel at ease", "I feel upset", "I am presently worrying over possible misfortunes", "I feel satisfied", "I feel frightened", "I feel comfortable", "I feel self-confident", "I feel nervous", "I am jittery", "I feel indecisive", "I am relaxed", "I feel content", "I am worried", "I feel confused", "I feel steady", "I feel pleasant". The statements had to be self-rated by the participants on a scale from 1 ("not at all") to 4 ("very much so"). Self-rated points were summarized according to the stencil of the STAI questionnaire, resulting in the summary score.

Behavioural assessments - ANT task. The ANT can be described as a modified version of the Erikson flanker task disentangling human attention networks.

In the flanker task, subjects are requested to respond immediately to the direction of a centrally presented arrow ($<$ or $>$) which is either directed to the right or left side by pressing a right or left button. The stimulus is randomly accompanied by additional arrows ('flankers'), which are presented in the same or opposite direction (congruent flankers, e.g. $< < < < <$, incongruent flankers, e.g. $> > < > >$) or no flankers appear (neutral condition, e.g. $<$).

When performing the ANT, the flankers are initialized by warning signals ('cues'): a 'center cue' provides time information about stimulus onset only, a cue above or below the central arrow provides time and stimulus placement information ('spatial cue'), simultaneously presented cues above and below the central arrow presents a prominent warning stimulus. Alternatively, no cue is presented ('no cue condition'). Subjects with the 'double cue's presented before the flanker stimulus onset show faster reaction times compared to the 'no cue' condition, known as the alerting effect. This effect is calculated by subtracting the reaction times (RT) of the 'double cue' and 'no cue condition'.

As expected, ANT results showed significant effects of cue ($p<0.001$), flanker ($p<0.001$), and cue by flanker interaction ($p<0.001$) in a mixed design ANOVA (factor cue with items no cue, center cue, spatial cue, double cue and factor flanker with items congruent, incongruent, neutral). Sidak corrected posthoc tests revealed the fastest reaction times in the spatial cue condition (RT 507ms), lowest in the nocue condition (RT 507ms) and doubleue/centercue condition (RT 534ms, RT541ms) in between (all three conditions differed $p<0.001$, whereas doublecue and nocue conditions did not differ significantly). Regarding the flanker condition, all flankers differed significantly in RT (neutral condition RT 475ms, congruent condition RT 509ms, incongruent RT 632ms, all $p<0.001$). Our ANT results are all in line with the results of previous publications.

MRI data acquisition. Structural image acquisition was performed with the following parameters for the anatomical scans using a Siemens MAGNETOM Prisma 3 T MRI scanner with syngo MR E11 software and a 64-channel head coil: 3D-MPRAGE sequence, echo time (TE) = 2.82 ms, repetition time (TR) = 2.500 ms, inversion time (T1) = 1.100 ms, flip angle = 7°, bandwidth = 140 Hz/pixel, acquisition volume = $256\times256\times192$ mm^3, isometric voxel size = 1.0 mm^3, scan duration = 5 min 18 s.

The acquisition of a neuromelanin sensitive sequence was performed with the following parameters using a Siemens MAGNETOM Prisma 3 T MRI scanner with syngo MR E11 software and a 64-channel head coil: Neuromelanin acts as an endogenous MR contrast agent: it shortens the longitudinal relaxation time T1 and the LC appears hyperintense in high-resolution T1-weighted MRI. We applied a T1-weighted TSE sequence according to the original work of Sasaki et al. (1) with the following imaging parameters: 14 axial slices, acquisition volume = $192\times192\times42$ mm^3, slice thickness = 2.50 mm, inter-slice gap 0.5 mm, leading to an effective slice thickness of 3 mm, TR = 634.0 ms, TE = 10.0 ms, bandwidth = 165 Hz/Pixel, flip angle = 80°, scan duration = 10 min 50s.

Functional MRI data were collected using a Siemens MAGNETOM 7 T MRI scanner with Siemens Syngo VB17 software and a 32-channel head coil using a multi-band accelerated T2*-weighted echo-planar imaging (EPI) sequence: 60 axial slices parallel to the anterior-posterior commissure plane covering the whole brain acquired in interleaved order, acquisition volume = $212\times212\times132$ mm^3, slice thickness = 2.0 mm, leading to a high resolution of 2.0 mm isotropic voxels and no gap, TR = 1500 ms, TE = 25.0 ms, flip angle = 70°, 400/500/500 volumes in total, scan duration = 10:00min/12:30min/12:30min. We visually checked raw data for each dataset and each time point. Scans one and two were performed on the same day, whereas the third scan was conducted on a separate session. The three scan sessions were concatenated for further analyses.

Both 3T MRI and 7T MRI scanners were used in the study: for the acquisition of the fMRI data, we could take advantage of high BOLD signal in 7T MRI (2), whereas for the DTI data acquisition, we especially profit from the excellent gradient strength of the 3T PRISMA MRI scanner.

Definition of individual LC location. The LC was manually segmented in the subjects by a radiologist. The method comprised delineation of the LC based on the neuromelanin contrast in FSLview (supplementary Figure 8, https://fsl.fmrib.ox.ac.uk/fsl/) and applying FSL FLIRT (https://fsl.fmrib.ox.ac.uk/fsl/) to interpolate the LC mask into functional or DTI space.

fMRI preprocessing. We started with the standard preprocessing pipeline implemented in CONN (https://www.conn-toolbox.org), which is based on SPM12 functions (https://www.fil.ion.ucl.ac.uk/spm/). Our steps included simultaneous realignment, unwarp and field map correction of the 7T functional data (as previously established at 3T and 7T MRI in (3, 4)), slice time correction (multiband sequence), ART based outlier detection (conservative settings with nuisance regression of time points exceeding the 95th percentile of movement compared to a normative sample to account for potential misplacement of LC, www.nitrc.org/projects/artifact_detect/). Then, the functional data were coregistered to the anatomical scans derived in the 3T MRI. The Freesurfer based segmented areas from DTI processing were then used as segmentation template to extract nuisance regressors from the functional data (supplementary Figure 7). Denoising included regression of white matter and CSF signals , removal of linear/quadratic trends, regression of subject motion (three-rotation and three-translation parameters and their first-order temporal derivatives), removal of motion outliers (scrubbing), and band-pass filtering at 0.008–0.09 Hz. The resting-state scans were concatenated to one experimental condition 'rest'. Then, the mean BOLD signals from the individual segmented LC regions were extracted within the unsmoothed EPI images and the timecourses were correlated to the extracted BOLD signal of the individual Freesurfer segmented brain areas in single-subject space (supplementary Figure 7). In other words, we gained individual LC functional connectivity matrices based on individual LC segmentation and individual segmentation of the targeted brain areas in every subject. In accordance to the processing of the DTI data, we thus performed a single subject analysis, avoiding loss of spatial accuracy compared to warping the EPI data into MNI space (5), and avoided smoothing, which could harm the spatial accuracy (6) of LC signal extraction gained with our subject specific delineation of the nucleus.

Statistical analysis. For intraclass correlation calculation, Generalizability Theory was applied. In G-theory, similar to the term 'factors' in parametric statistics, the term 'facets' is used to describe sources of variance in the data, and G-theory estimates the degree of variance attributable to those facets – in our case the individual subjects' fMRI or DTI measurements.

Generalizability coefficients (G-coefficients) and dependability coefficients (D-coefficients) are then computed with respect to variances of the defined data. Similar to the intraclass-correlation coefficient (ICC), the 'G-coefficient' represents the (ICC average) score of variance within all the facets (the overall variance of all subjects' DTI or fMRI runs), and the 'D-coefficient' the variance of a chosen number of facets (e.g. the overall LC DTI connectivity variance of one subject). Superior to ICC statistics, the D-coefficient can be created for any number of subjects, and in our case, the D-score of one subject within the overall G results was the focused measure to judge if it is reasonable - in respect to the overall data variance between all subjects' runs - to make inferences based on a single subjects' LC DTI or fMRI result only (similar to ICC "single observation"). G and D coefficients for functional MRI and DTI were assessed within a single-facet fully-crossed design. We then constructed a two-facet nested design with the facets 'subjects DTI' and 'subjects fMRI' and the items brain regions as for the separate analyses to investigate the pairwise overall similarity between the two measures.

Data illustrations. For the funtional data in Figures 1 and 2b, we applied a threshold of z=0.15 to emphasize the stronger connections. For the structural data, no thresholds were used because of the application of SIFT2. In Figure 2b, we used the F-values of the one-sample permutation test (FDR<0.05) and applied the results matrix within the anatomy of our example participant to present group statistics of structural connectivity (same participant as in Figure 1). For the group statistics of functional connectivity, the presented values are h-values, which corresponds to the average Fischer-transformed pairwise correlations of the specified contrast (one-sample t-test, presented brain regions do not exceed FDR<0.05).

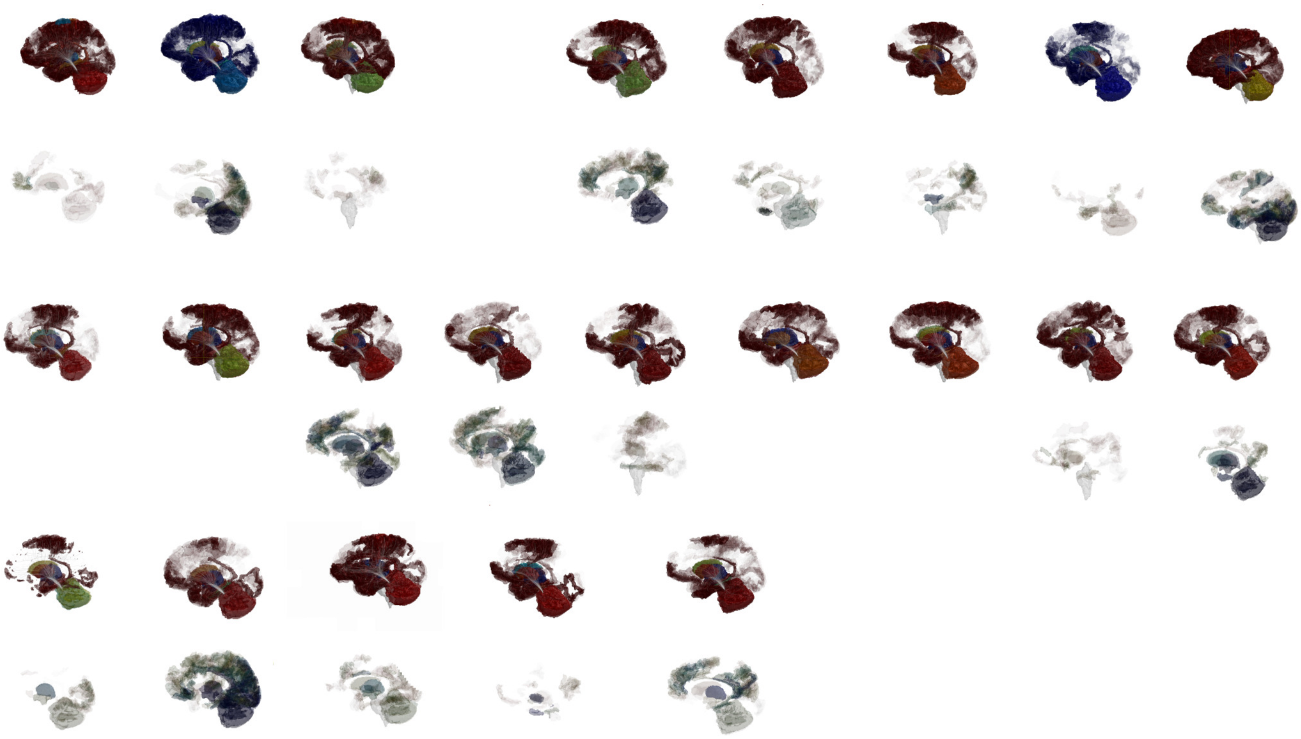

Fig. S1. Raw values of structural (first rows) and corresponding functional (second rows) locus coeruleus connectivity of the participants investigated in this study. All visualized regions represent the individual segmented brain anatomy of the subjects. Color-coding was performed according to Figure 1 in the original paper, with the participant presented in Figure 1 left out in this illustration (fourth participant, upper rows). The functional connectivity of four participants were excluded due to bad registration (middle rows, see methods section).

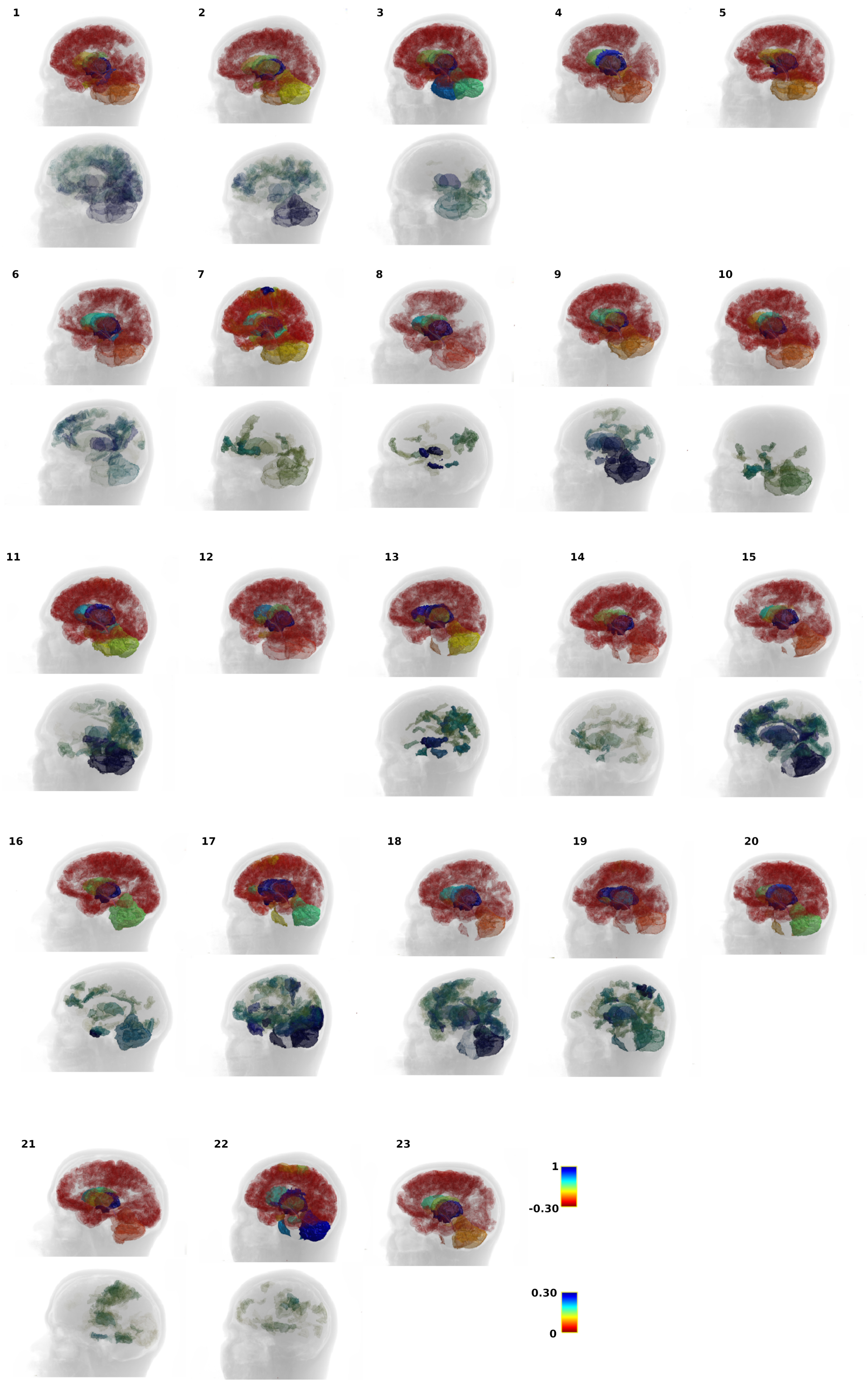

Fig. S2. Illustration of all 23 subjects investigated in this study. The upper rows represent DTI structural LC connectivity (SIFT2, z-transformed values). The lower rows represent corresponding 7T LC resting-state, single subject space functional connectivity (35min resting-state fMRI, n=18 due to data exclusions).

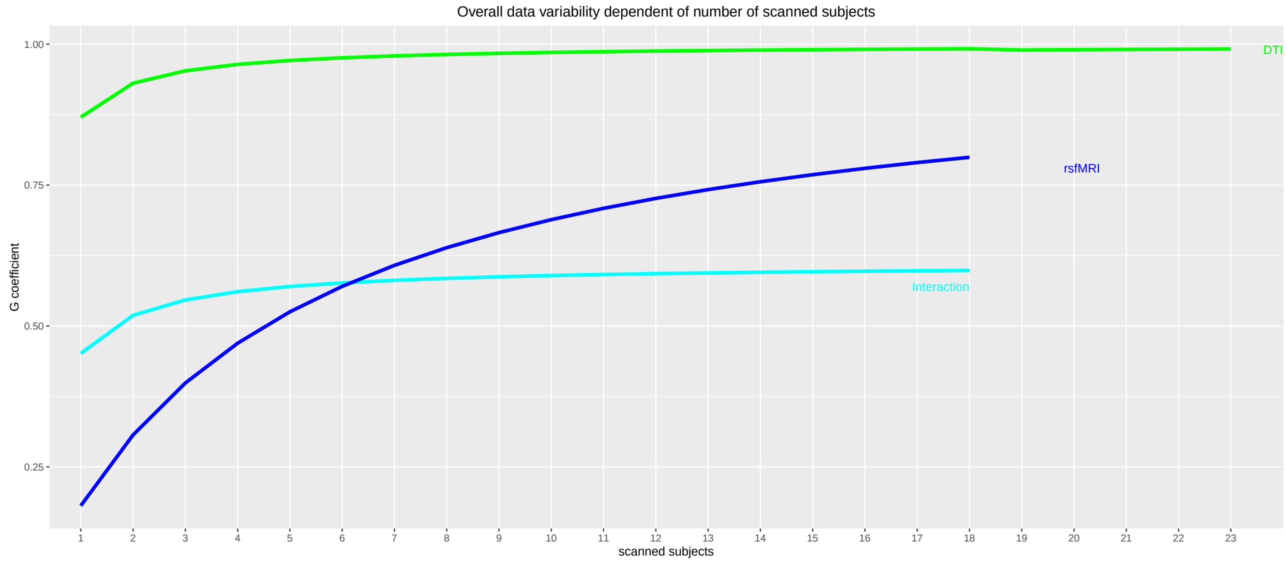

Fig. S3. G-coefficients of locus coeruleus functional connectivity (rsfMRI) and locus coeruleus structural connectivity (DTI) and their interaction term (interaction) dependent of number of scanned subjects calculated by corresponding D-studies.

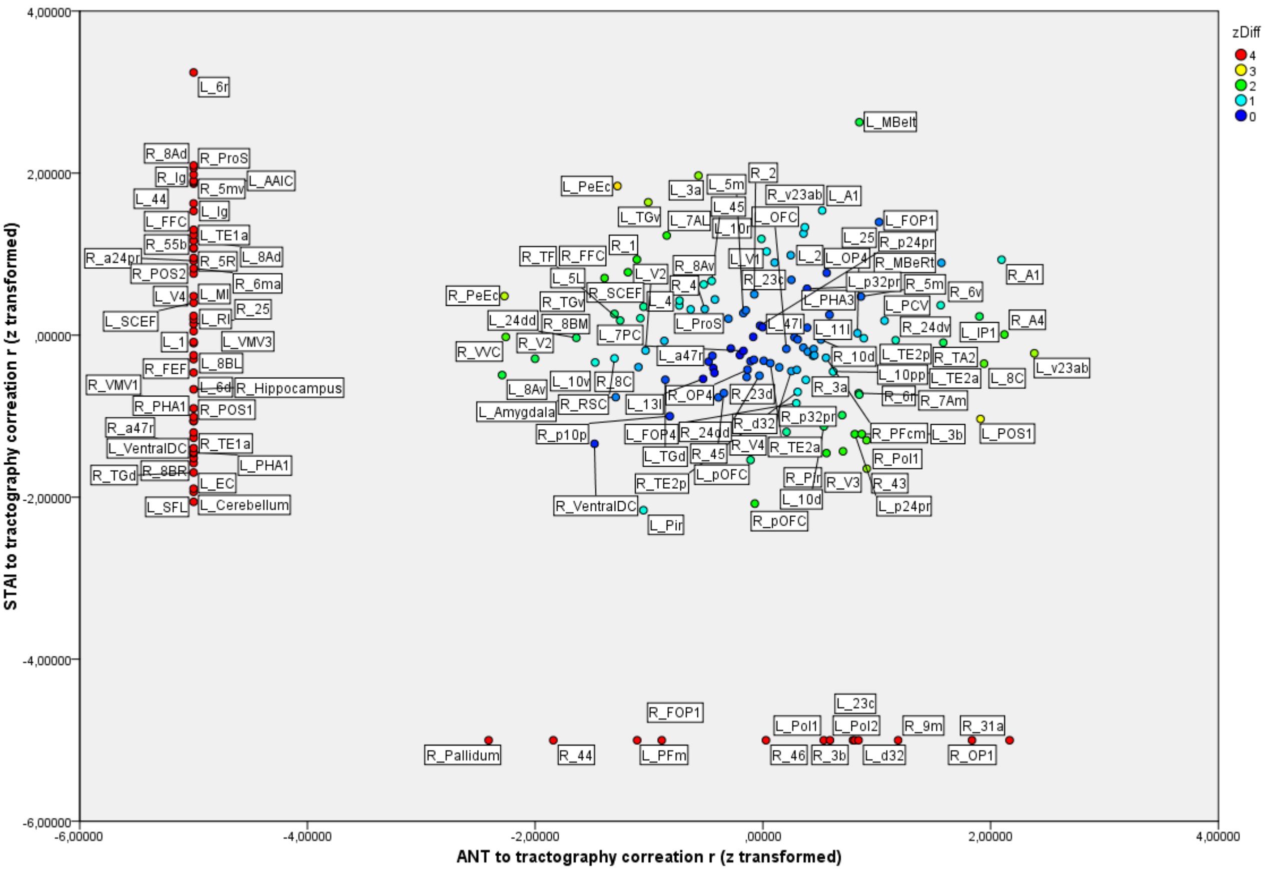

Fig. S4. Illustration of the relationship of z-transformed r-correlation coefficients between LC structural connectivity (fiber count) and ANT/STAI scores. The red points near the axes represent areas that show a relationship to ANT (x axis) or STAI scores (y axis) only, whereas the blueish areas in the middle correlate to both ANT and STAI scores. The color represents the difference or in other words the distance of the z-transformed r correlation coefficients between the ANT and STAI groups (zDiff). The areas with the high zDiff scores (green to yellow) at the borders of the middle pulk correlate stronger to ANT (nearer to x axis) or to STAI (nearer to y axis), respectively. For example, the LC structural connectivity to areas L-PeEc and R-PeEc correlate both to ANT and STAI scores, but much stronger to STAI scores then to ANT scores, whereas LC to R_Hippocampus structural connectivity correlates with STAI scores only and LC to L_PoI1 and LC to L_PoI2 correlate with ANT scores only. The list of area names associated with the abbreviations used here can be found in supplementary Table 3.

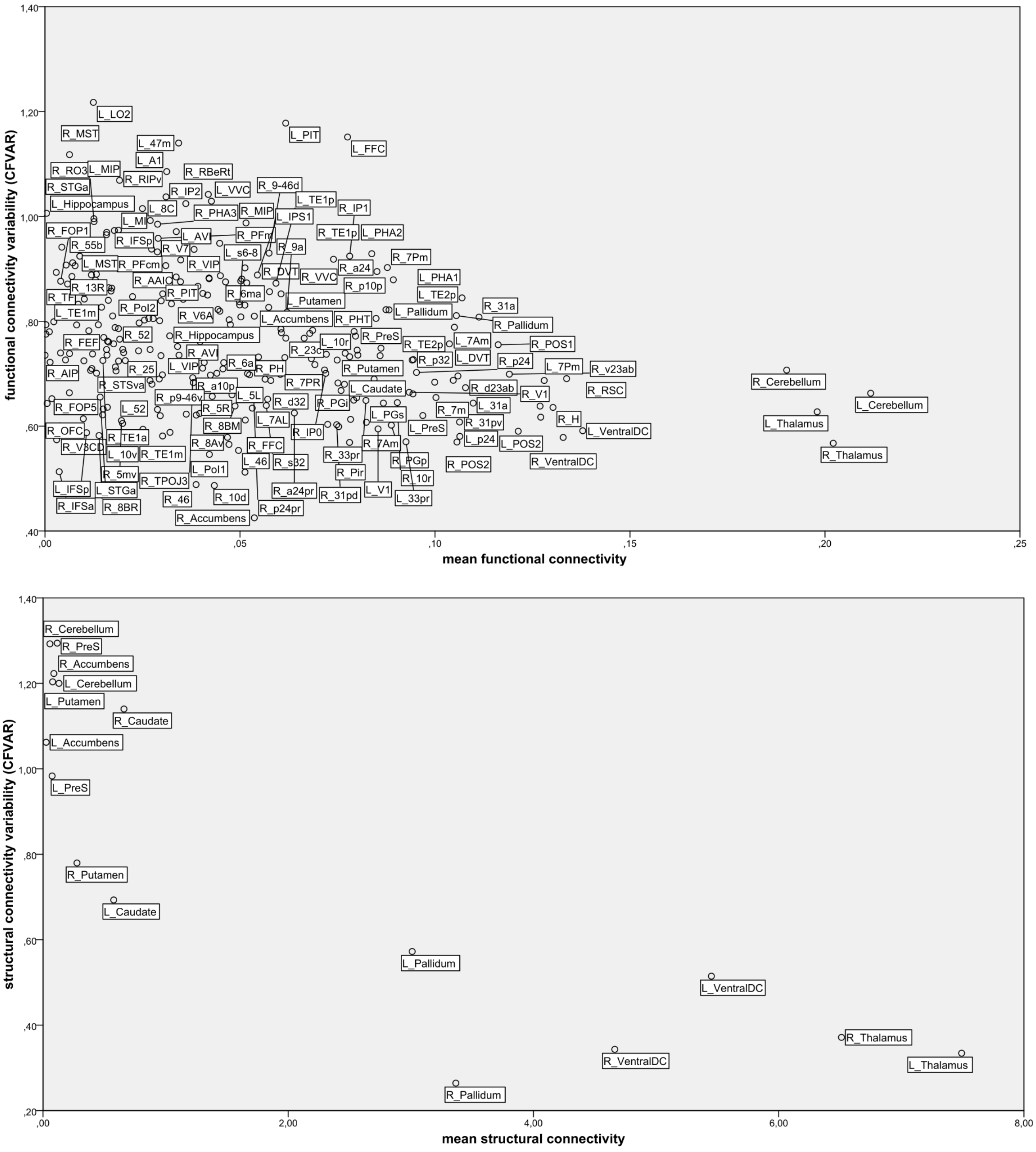

Fig. S5. Dependence of functional (upper panel) and structural (lower panel) connectivity variability (CFVAR) across individuals on connectivity strength (mean values across individuals), respectively. Both functional and structural connectivity variability (the coefficient of variation – calculated by the standard deviation divided by the mean) is linearly indirectly dependent on connectivity strength (Spearman two-sided correlation coefficient, p<0.001 for both panels). The list of area names associated with the abbreviations used here can be found in supplementary Table 3.

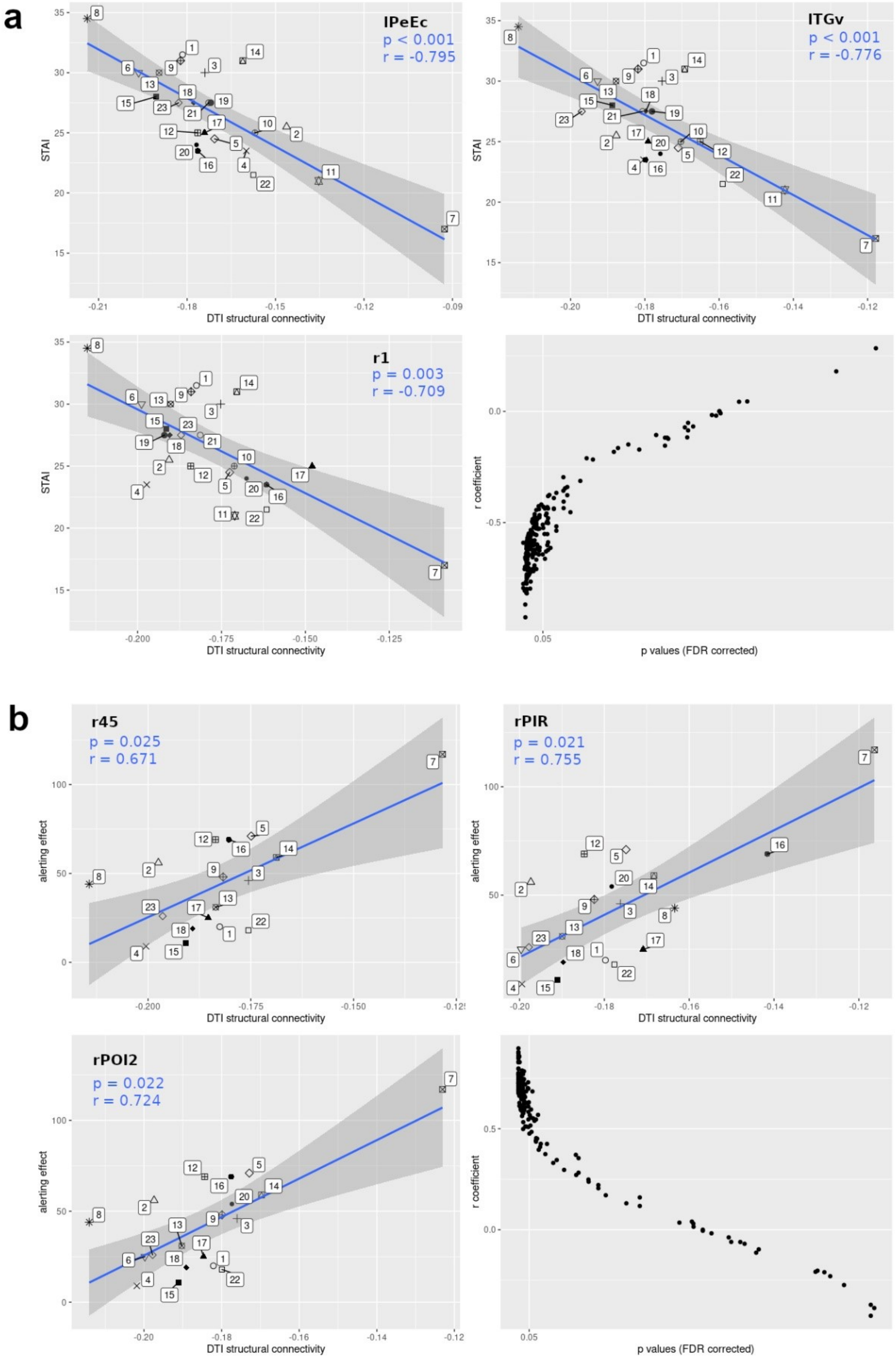

Fig. S6. Illustration of exemplary brain regions, which correlate in their structural connectivity to the LC with anxiety (a) and alertness (b) scores (upper panels and lower left panels). The symbols representing single subjects' scores are in accordance with subject symbols of Figure 3 in the main paper. The lower right panels provide an overview over the correlation of all brain regions, and show that the majority of brain regions is significantly correlated to the respective score (a, anxiety; b, alertness; p<0.05, FDR corrected).

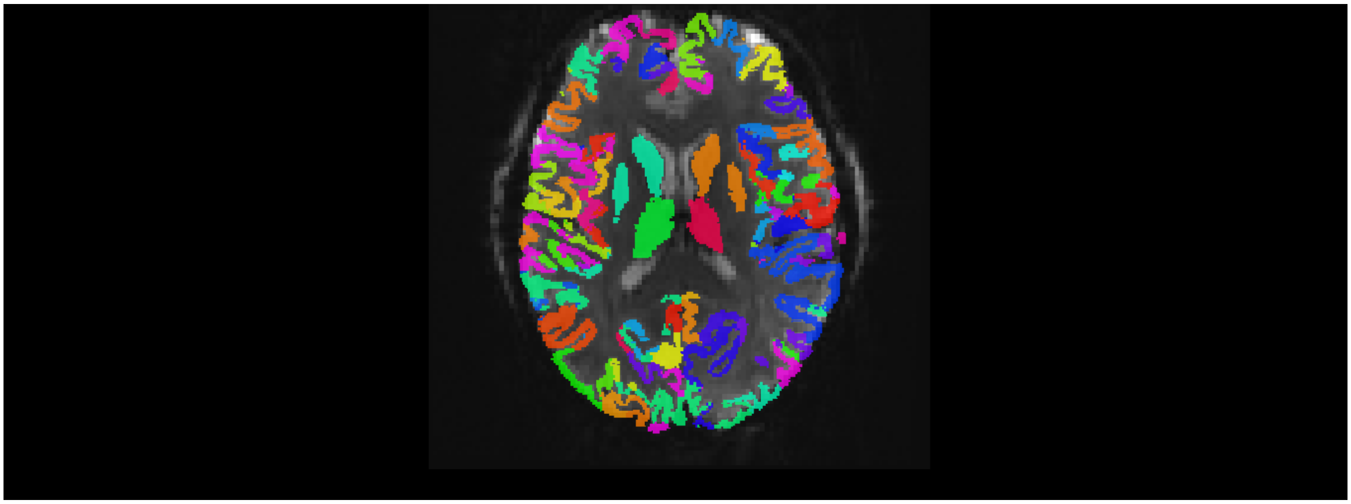

Fig. S7. Exemplary T1 anatomical-image based Freesurfer segmentation/Glasser parcellation of the brain overlayed on the subjects' 7T fMRI data, which was registered to the single subject anatomical image. The same parcellation scheme within subject space was used for both structural and functional connectome calculation.

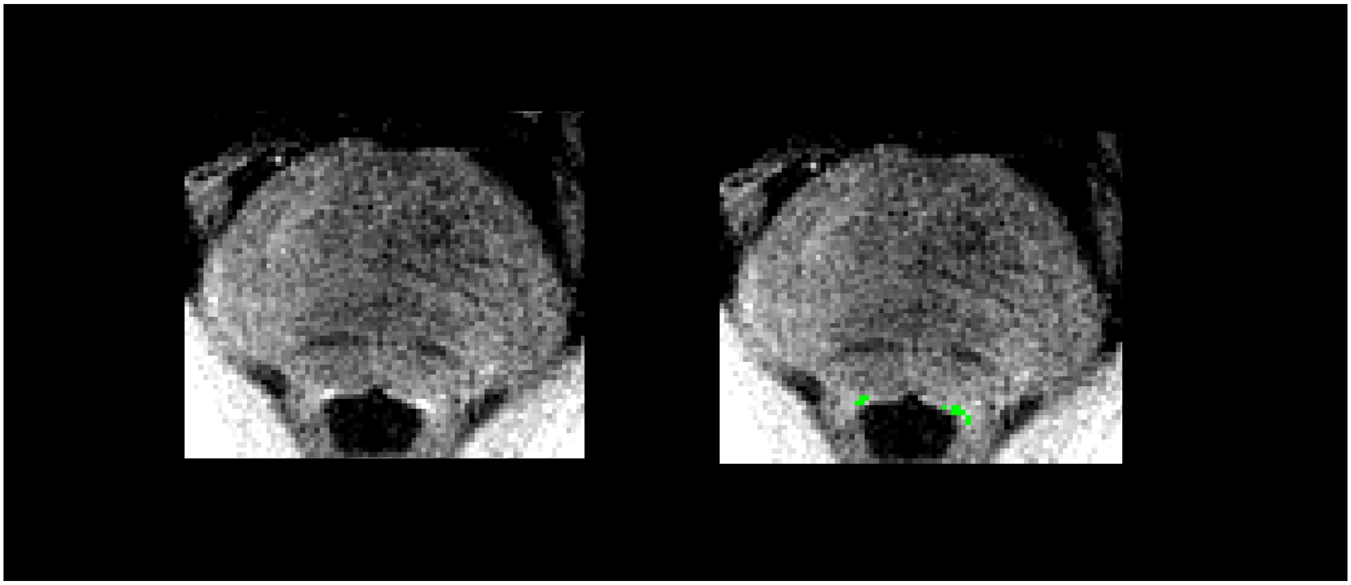

Fig. S8. The neuromelanin sensitive TSE sequence uncovers the locus coeruleus within transversal slices of the pons. The LC was manually delineated (two green spots at the ventral boarder of the IVth ventricle).

Table S1. Statistics of structural (DTI) and functional connectivity (fc) of the locus coeruleus (all regions significant to FDR<0.05) on the group level.

DTI<br>Region	Regionnames	F-Statistic	rsFC<br>Region	Regionnames	beta	T-statistic
L__Thalamus	Thalamus	8.0376	L__Cerebellum	Cerebellum	0.211721	6.402246
L__VentralDC	VentralDC	7.3367	R__Thalamus	Thalamus	0.202124	7.478475
R__Thalamus	Thalamus	6.6509	L__Thalamus	Thalamus	0.198008	6.763312
R__VentralDC	VentralDC	6.2874	R__Cerebellum	Cerebellum	0.190179	6.002420
R__Pallidum	Pallidum	4.8520	L__VentralDC	VentralDC	0.137883	3.722569
L__Pallidum	Pallidum	4.2450	R__RSC	RetroSpleniaComplex	0.133745	6.140235
R__Caudate	Caudate	0.8137	R__VentralDC	VentralDC	0.132869	4.076012
L__Caudate	Caudate	0.7777	R__H	Hippocampus	0.130263	6.039088
R__Putamen	Putamen	0.6150	L__7Pm	MediaArea__7P	0.128006	5.781079
L__Putamen	Putamen	0.2868	L__POS1	Parieto-OccipitaSulcus__Area__1	0.127115	6.875808
R__Accumbens	Accumbens	0.2744	L__RSC	RetroSpleniaComplex	0.126943	6.642794
R__PreS	PreSubiculum	0.2323	L__POS2	Parieto-OccipitaSulcus__Area__2	0.121342	6.488893
L__Accumbens	Accumbens	0.2041	R__v23ab	Area__ventra23__a+b	0.119069	4.495428
L__pOFC	PosterioOFC__Complex	0.1957	R__POS1	Parieto-OccipitaSulcus__Area__1	0.116201	5.140125
L__PreS	PreSubiculum	0.1823	R__31a	Area__31a	0.111291	5.156674
L__Cerebellum	Cerebellum	0.1717	L__31a	Area__31a	0.109793	5.289231
R__EC	EntorhinaCortex	0.1669	R__d23ab	Area__dorsa23__a+b	0.107829	4.654173
R__Cerebellum	Cerebellum	0.1663	L__TE2p	Area__TE2__posterior	0.106934	3.850248
L__10pp	Pola10p	0.1470	L__p24	Area__posterio24	0.106363	4.880209
L__6mp	Area__6mp	0.1413	R__31pv	Area__31p__ventral	0.106299	4.972342
L__6ma	Area__6m__anterior	0.1313	L__DVT	DorsaTransitionaVisuaArea	0.105880	4.367159
R__6ma	Area__6m__anterior	0.1272	R__POS2	Parieto-OccipitaSulcus__Area__2	0.105626	5.370430
R__SFR	SuperioFrontalanguage__Area__R	0.1106	R__Pallidum	Pallidum	0.105474	4.410118
L__EC	EntorhinaCortex	0.1025	L__Pallidum	Pallidum	0.104982	4.323886
L__SFL	SuperioFrontalanguage__Area	0.0925	L__v23ab	Area__ventra23__a+b	0.104680	5.104994
R__Amygdala	Amygdala	0.0897	L__7Am	MediaArea__7A	0.103690	3.891178
R__10pp	Pola10p	0.0792	R__7m	Area__7m	0.100251	4.722261
L__10v	Area__10v	0.0632	L__7m	Area__7m	0.099931	5.357216
R__6mp	Area__6mp	0.0610	L__PreS	PreSubiculum	0.096878	6.486013
R__6d	Dorsaarea__6	0.0594	R__p24	Area__posterio24	0.095254	3.619465
R__4	Primary__MotoCortex	0.0568	R__p32	Area__p32	0.094438	3.830806
L__PeEc	PerirhinaEctorhinaCortex	0.0559	L__a32pr	Area__anterio32__prime	0.094347	5.836892
L__H	Hippocampus	0.0550	L__PCV	PreCuneus__VisuaArea	0.094146	5.430610
L__TGv	Area__TG__Ventral	0.0547	R__8Ad	Area__8Ad	0.093760	4.508624
L__SCEF	Supplementary__and__Cingulate__Eye__Field	0.0526	R__V1	Primary__VisuaCortex	0.093382	3.744288
R__SCEF	Supplementary__and__Cingulate__Eye__Field	0.0518	L__33pr	Area__33__prime	0.092558	4.242231
R__TGd	Area__TG__dorsal	0.0507	R__10r	Area__10r	0.090344	3.550428
L__OFC	OrbitaFrontaComplex	0.0469	L__PHA1	ParaHippocampaArea__1	0.089315	4.182062
R__TGv	Area__TG__Ventral	0.0459	R__PGp	Area__PGp	0.088812	4.137858
R__PeEc	PerirhinaEctorhinaCortex	0.0436	R__p10p	Area__posterio10p	0.088209	4.104556
L__TGd	Area__TG__dorsal	0.0420	R__7Pm	MediaArea__7P	0.087790	3.939781
R__s6-8	Superio6-8__TransitionaArea	0.0413	L__IP1	Area__IntraParieta1	0.087427	4.452228
L__6d	Dorsaarea__6	0.0343	L__PGs	Area__PGs	0.086773	5.186056
L__1	Area__1	0.0332	L__PGp	Area__PGp	0.086149	4.814878
R__H	Hippocampus	0.0292	R__a32pr	Area__anterio32__prime	0.085940	3.833669
L__RSC	RetroSpleniaComplex	0.0291	R__TE2p	Area__TE2__posterior	0.085921	3.887479
R__5R	Area__5R	0.0276	L__V1	Primary__VisuaCortex	0.085403	3.826045
L__4	Primary__MotoCortex	0.0275	R__a24	Area__a24	0.085116	3.185550
R__a47r	Area__anterio47r	0.0266	L__7PL	LateraArea__7P	0.084883	3.457973
L__11l	Area__11l	0.0256	L__d23ab	Area__dorsa23__a+b	0.084397	4.082069
R__8BR	Area__8BateraR	0.0254	L__PHA2	ParaHippocampaArea__2	0.083791	3.593760
R__a10p	Area__anterio10p	0.0244	L__8Ad	Area__8Ad	0.082880	4.315698
R__1	Area__1	0.0243	R__7Am	MediaArea__7A	0.082397	3.148575
L__5L	Area__5L	0.0237	R__33pr	Area__33__prime	0.082293	3.782757
R__8BM	Area__8BM	0.0235	L__10r	Area__10r	0.080224	4.316710
L__i6-8	Inferio6-8__TransitionaArea	0.0225	R__PreS	PreSubiculum	0.080037	2.709416
R__47R	Area__47(47ateral)__R	0.0223	R__23d	Area__23d	0.079934	4.119655
L__Amygdala	Amygdala	0.0208	R__PGs	Area__PGs	0.079890	4.969030
R__IFSp	Area__IFSp	0.0188	L__IP0	Area__IntraParieta0	0.079627	3.571657
L__a47r	Area__anterio47r	0.0184	R__PHT	Area__PHT	0.079260	3.986464
R__Pir	Pirform__Cortex	0.0176	R__PGi	Area__PGi	0.079168	4.896762
R__7Am	MediaArea__7A	0.0175	L__a24pr	Anterio24__prime	0.078356	4.141617
R__33pr	Area__33__prime	0.0166	R__IP1	Area__IntraParieta1	0.078124	3.844501
R__6a	Area__6__anterior	0.0161	R__Putamen	Putamen	0.078119	3.621470
L__7AL	LateraArea__7A	0.0151	R__Pir	Pirform__Cortex	0.078092	2.958943
R__7AR	LateraArea__7A__R	0.0151	R__s6-8	Superio6-8__TransitionaArea	0.076968	3.551718
R__i6-8	Inferio6-8__TransitionaArea	0.0151	L__Caudate	Caudate	0.076777	3.267484
R__p47r	Area__posterio47r	0.0151	L__a24	Area__a24	0.075909	3.195119
L__6a	Area__6__anterior	0.0132	L__23c	Area__23c	0.075300	4.662182
R__RSC	RetroSpleniaComplex	0.0129	L__23d	Area__23d	0.075125	3.844143
L__Pir	Pirform__Cortex	0.0128	L__31pv	Area__31p__ventral	0.074911	3.867590
R__p9-46v	Area__posterio9-46v	0.0123	R__31pd	Area__31pd	0.074793	3.709854
L__47l	Area__47(47ateral)	0.0116	R__PCV	PreCuneus__VisuaArea	0.074054	3.624502
R__FEF	FrontaEye__Fields	0.0116	R__TE1p	Area__TE1__posterior	0.073977	3.217973
R__OFC	OrbitaFrontaComplex	0.0116	L__i6-8	Inferio6-8__TransitionaArea	0.072490	3.516268
L__a10p	Area__anterio10p	0.0113	L__p32	Area__p32	0.072133	3.650317
L__8BL	Area__8Bateral	0.0110	R__IP0	Area__IntraParieta0	0.072002	3.446858
R__pOFC	PosterioOFC__Complex	0.0108	R__Caudate	Caudate	0.071681	2.858346
R__24dd	DorsaArea__24d	0.0102	L__H	Hippocampus	0.069200	3.552129
L__24dd	DorsaArea__24d	0.0101	L__Putamen	Putamen	0.068634	3.309955
L__V6A	Area__V6A	0.0101	L__31pd	Area__31pd	0.068292	3.679125
R__3b	Primary__Sensory__Cortex	0.0101	L__p10p	Area__posterio10p	0.067891	3.113558
L__PoI2	PosterioInsulaArea__2	0.0100	L__PHA3	ParaHippocampaArea__3	0.066456	2.953719
L__3b	Primary__Sensory__Cortex	0.0098	R__a24pr	Anterio24__prime	0.063935	3.552865
R__VIP	VentraIntraParietaComplex	0.0097	R__9a	Area__9__anterior	0.062042	3.086525
L__DVT	DorsaTransitionaVisuaArea	0.0094	R__23c	Area__23c	0.061749	3.064299
L__PGp	Area__PGp	0.0094	R__PH	Area__PH	0.061496	3.068593
R__23d	Area__23d	0.0094	R__PHA2	ParaHippocampaArea__2	0.060615	3.261888
L__7PL	LateraArea__7P	0.0091	R__VVC	VentraVisuaComplex	0.060526	2.542145
L__7PC	Area__7PC	0.0091	L__Accumbens	Accumbens	0.060493	3.435773
R__8Av	Area__8Av	0.0089	L__TE1p	Area__TE1__posterior	0.059221	2.459143
R__10d	Area__10d	0.0089	R__d32	Area__dorsa32	0.057901	2.502713
R__44	Area__44	0.0085	R__A1	Primary__Auditory__Cortex	0.057571	2.351958
R__p10p	Area__posterio10p	0.0084	R__9-46d	Area__9-46d	0.057449	2.700067
L__VIP	VentraIntraParietaComplex	0.0081	L__IP2	Area__IntraParieta2	0.057355	3.586872
L__10d	Area__10d	0.0078	L__p32pr	Area__p32__prime	0.057088	3.041397
R__5m	Area__5m	0.0078	R__s32	Area__s32	0.056736	2.381451
R__PHA1	ParaHippocampaArea__1	0.0078	L__PH	Area__PH	0.056441	3.583349
L__47s	Area__47s	0.0075	L__p9-46v	Area__posterio9-46v	0.054743	2.815916
R__45	Area__45	0.0074	L__IPS1	IntraParietaSulcus__Area__1	0.054461	2.916626
L__7Am	MediaArea__7A	0.0072	R__Accumbens	Accumbens	0.053679	2.847005
R__11R	Area__11R	0.0072	L__5L	Area__5L	0.052466	3.146927
R__10v	Area__10v	0.0066	L__p24pr	Area__Posterio24__prime	0.051923	2.807760

Table S1 continued from previous page

DTI Region	Regionnames	F-Statistic	rsFC Region	Regionnames	beta	T-statistic
L__FEF	FrontaEye__Fields	0.0063	L__6ma	Area__6m__anterior	0.051358	2.905222
R__6v	VentraArea__6	0.0063	L__9-46d	Area__9-46d	0.051282	2.634079
R__IFSa	Area__IFSa	0.0063	R__TPOJ2	Area__TemporoParietoOccipitaJunction__2	0.051247	2.625499
R__PGp	Area__PGp	0.0062	R__RIPd	RIPd	0.050469	3.366830
R__8C	Area__8C	0.0060	R__6ma	Area__6m__anterior	0.050191	2.613918
R__V6A	Area__V6A	0.0060	L__s6-8	Superio6-8__TransitionaArea	0.049905	2.884244
L__33pr	Area__33__prime	0.0058	R__FST	Area__FST	0.049831	3.165279
L__s6-8	Superio6-8__TransitionaArea	0.0058	L__46	Area__46	0.049624	2.808892
L__PoI1	Area__PosterioInsula1	0.0056	L__LIPd	AreaateraIntraParietadorsal	0.048919	3.741452
L__8BM	Area__8BM	0.0055	R__8Av	Area__8Av	0.048721	3.147021
R__24dv	VentraArea__24d	0.0054	R__i6-8	Inferio6-8__TransitionaArea	0.047532	2.734987
L__55b	Area__55b	0.0053	L__a9-46v	Area__anterio9-46v	0.047175	2.837231
L__V3CD	Area__V3CD	0.0050	R__PHA1	ParaHippocampaArea__1	0.046293	2.702932
R__p32pr	Area__p32__prime	0.0050	R__6a	Area__6__anterior	0.045835	2.483658
L__8Av	Area__8Av	0.0049	L__FST	Area__FST	0.044356	2.773186
R__a9-46v	Area__anterio9-46v	0.0049	R__a10p	Area__anterio10p	0.044056	2.714958
R__PoI2	PosterioInsulaArea__2	0.0049	L__5mv	Area__5m__ventral	0.042409	2.836156
R__46	Area__46	0.0048	L__PoI1	Area__PosterioInsula1	0.042149	2.429049
L__5m	Area__5m	0.0046	R__p9-46v	Area__posterio9-46v	0.041817	2.444165
L__9m	Area__9__Middle	0.0046	L__a10p	Area__anterio10p	0.041584	2.737901
L__V2	Second__VisuaArea	0.0044	R__RI	RetroInsulaCortex	0.033623	2.679894
R__7PC	Area__7PC	0.0044				
L__FOP4	FrontaOperculaArea__4	0.0042				
L__TE2a	Area__TE2__anterior	0.0041				
R__AAIC	AnterioAgranulaInsula__Complex	0.0040				
L__TF	Area__TF	0.0039				
L__PGs	Area__PGs	0.0039				
R__5mv	Area__5m__ventral	0.0039				
R__7PR	LateraArea__7P__R	0.0039				
R__55b	Area__55b	0.0038				
R__23c	Area__23c	0.0036				
R__47s	Area__47s	0.0034				
L__V3	Third__VisuaArea	0.0033				
L__V4	Fourth__VisuaArea	0.0033				
R__V2	Second__VisuaArea	0.0033				
L__LIPv	AreaateraIntraParietaventral	0.0032				
R__9a	Area__9__anterior	0.0032				
R__PCV	PreCuneus__VisuaArea	0.0031				
R__TF	Area__TF	0.0031				
R__V3A	Area__V3A	0.0030				
R__3a	Area__3a	0.0030				
R__9p	Area__9__Posterior	0.0030				
R__6r	RostraArea__6	0.0030				
L__45	Area__45	0.0029				
R__TE2a	Area__TE2__anterior	0.0029				
L__AAIC	AnterioAgranulaInsula__Complex	0.0027				
L__V3A	Area__V3A	0.0026				
L__8Ad	Area__8Ad	0.0026				
L__TE2p	Area__TE2__posterior	0.0025				
L__p10p	Area__posterio10p	0.0025				
L__POS2	Parieto-OccipitaSulcus__Area__2	0.0024				
L__13l	Area__13l	0.0024				
R__DVT	DorsaTransitionaVisuaArea	0.0024				
L__V1	Primary__VisuaCortex	0.0023				
L__p32pr	Area__p32__prime	0.0023				
L__FOP1	FrontaOperculaArea__1	0.0023				
R__FOP1	FrontaOperculaArea__1	0.0023				
L__PCV	PreCuneus__VisuaArea	0.0022				
R__V1	Primary__VisuaCortex	0.0022				
R__VMV1	VentroMediaVisuaArea__1	0.0022				
L__24dv	VentraArea__24d	0.0021				
R__V3	Third__VisuaArea	0.0021				
R__TE2p	Area__TE2__posterior	0.0021				
R__43	Area__43	0.0020				
R__31a	Area__31a	0.0020				
L__ProS	ProStriate__Area	0.0019				
L__PHA1	ParaHippocampaArea__1	0.0019				
L__PHA3	ParaHippocampaArea__3	0.0019				
R__RO2	RO2	0.0019				
R__47m	Area__47m	0.0019				
L__7Pm	MediaArea__7P	0.0018				
L__2	Area__2	0.0018				
L__IP1	Area__IntraParieta1	0.0018				
L__d32	Area__dorsa32	0.0017				
L__31a	Area__31a	0.0017				
R__25	Area__25	0.0017				
L__6v	VentraArea__6	0.0016				
R__V7	Seventh__VisuaArea	0.0016				
R__p24pr	Area__Posterio24__prime	0.0016				
R__VVC	VentraVisuaComplex	0.0016				
R__PoI1	Area__PosterioInsula1	0.0016				
L__PFm	Area__PFm__Complex	0.0015				
R__d32	Area__dorsa32	0.0015				
R__OP4	Area__OP4-PV	0.0015				
L__6r	RostraArea__6	0.0014				
L__MI	Middle__InsulaArea	0.0014				
R__STGa	Area__STGa	0.0014				
R__PGs	Area__PGs	0.0014				
L__IP0	Area__IntraParieta0	0.0013				
L__VVC	VentraVisuaComplex	0.0013				
R__2	Area__2	0.0013				
R__PF	Area__PF__Complex	0.0013				
L__PF	Area__PF__Complex	0.0012				
R__9m	Area__9__Middle	0.0012				
R__TE1a	Area__TE1__anterior	0.0012				
R__IP1	Area__IntraParieta1	0.0012				
R__A4	Auditory__4__Complex	0.0012				
L__23d	Area__23d	0.0011				
R__MIP	MediaIntraParietaArea	0.0011				
R__9-46d	Area__9-46d	0.0011				
R__TE1p	Area__TE1__posterior	0.0011				
R__PHT	Area__PHT	0.0011				
R__PFop	Area__PF__Opercular	0.0011				
R__p24	Area__posterio24	0.0011				
L__FFC	Fusiform__Face__Complex	0.0010				
R__V4	Fourth__VisuaArea	0.0010				
R__POS2	Parieto-OccipitaSulcus__Area__2	0.0010				

Table S1 continued from previous page

DTI Region	Regionnames	F-Statistic	rsFC Region	Regionnames	beta	T-statistic
R__PIT	PosterioInferoTemporacomplex	0.0010				
L__v23ab	Area__ventra23__a+b	0.0009				
L__23c	Area__23c	0.0009				
L__10r	Area__10r	0.0009				
L__OP1	Area__OP1-SII	0.0009				
R__MT	Middle__TemporaArea	0.0009				
L__p24pr	Area__Posterio24__prime	0.0008				
L__9p	Area__9__Posterior	0.0008				
L__46	Area__46	0.0008				
R__v23ab	Area__ventra23__a+b	0.0008				
R__8Ad	Area__8Ad	0.0008				
L__LO2	AreaateraOccipita2	0.0007				
L__d23ab	Area__dorsa23__a+b	0.0007				
L__8C	Area__8C	0.0007				
L__25	Area__25	0.0007				
L__A4	Auditory__4__Complex	0.0007				
R__FFC	Fusiform__Face__Complex	0.0007				
R__TA2	Area__TA2	0.0007				
R__ProS	ProStriate__Area	0.0007				
R__PFm	Area__PFm__Complex	0.0007				
L__STGa	Area__STGa	0.0006				
L__PHT	Area__PHT	0.0006				
R__13R	Area__13R	0.0006				
R__PH	Area__PH	0.0006				
R__V4t	Area__V4t	0.0006				
R__TE1m	Area__TE1__Middle	0.0006				
L__a9-46v	Area__anterio9-46v	0.0005				
L__p47r	Area__posterio47r	0.0005				
R__PFcm	Area__PFcm	0.0005				
R__V3CD	Area__V3CD	0.0005				
L__TE1a	Area__TE1__anterior	0.0004				
L__V4t	Area__V4t	0.0004				
R__PSR	PeriSylviananguage__Area__R	0.0004				
R__PGi	Area__PGi	0.0004				
L__TE1p	Area__TE1__posterior	0.0003				
R__A5	Auditory__5__Complex	0.0002				

Table S2. Relationship of Alertness (ANT) scores and STAI (anxiety) scores to LC structural connectivity. The numbers represent r-coefficients of the brain regions, which show significant correlation between structural LC connectivity and alertness/anxiety (all regions significant to FDR<0.05).

Regions	Regionnames	Alertness	STAI
L__V1	Primary__Visual__Cortex__L	0.70782345	-0.705866989
L__MST			
L__V6			
L__V2	Second__Visual__Area__L	0.6134167157	-0.6038861217
L__V3	Third__Visual__Area__L	0.6824135322	-0.5986895926
L__V4	Fourth__Visual__Area__L		-0.6669886098
L__V8			
L__4	Primary__Motor__Cortex__L	0.6381149976	-0.656137907
L__3b	Primary__Sensory__Cortex__L	0.7715376925	-0.5069963193
L__FEF			
L__PEF			
L__55b			
L__V3A			
L__RSC	RetroSplenial__Complex__L	0.6629715893	-0.5835596738
L__POS2			
L__V7			
L__IPS1			
L__FFC	Fusiform__Face__Complex__L		-0.7380834593
L__V3B			
L__LO1			
L__LO2			
L__PIT			
L__MT			
L__A1	Primary__Auditory__Cortex__L	0.742585258	-0.7663277036
L__PSL			
L__SFL	Superior__Frontal__Language__Area__L		-0.4398596731
L__PCV	PreCuneus__Visual__Area__L	0.7882175445	-0.6385128274
L__STV			
L__7Pm			
L__7m			
L__POS1	Parieto-Occipital__Sulcus__Area__1__L	0.8583827394	-0.5245103098
L__23d	Area__23d__L	0.674006493	-0.6407727121
L__v23ab	Area__ventral__23__a+b__L	0.8977850677	-0.600625669
L__d23ab			
L__31pv			
L__5m	Area__5m__L	0.6849342306	-0.6471766296
L__5mv			
L__23c	Area__23c__L	0.7663144164	
L__5L	Area__5L__L	0.5834393484	-0.6878963183
L__24dd	Dorsal__Area__24d__L	0.5627806992	-0.6186229203
L__24dv	Ventral__Area__24d__L	0.6271215342	-0.6150204243
L__7AL	Lateral__Area__7A__L	0.6289409597	-0.7372958877
L__SCEF	Supplementary__and__Cingulate__Eye__Field__L		-0.6593121825
L__6ma	Area__6m__anterior__L	0.6595900658	-0.591004895
L__7Am	Medial__Area__7A__L	0.7363679684	-0.6056918137
L__7PL			
L__7PC	Area__7PC__L	0.6849767384	-0.6034840251
L__LIPv			
L__VIP			
L__MIP			
L__1	Area__1__L		-0.613732061
L__2	Area__2__L	0.7195850314	-0.7143704048
L__3a	Area__3a__L	0.6521230918	-0.8067262877
L__6d	Dorsal__area__6__L		-0.5782354237
L__6mp	Area__6mp__L		-0.6401258696
L__6v			
L__p24pr	Area__Posterior__24__prime__L	0.7664839761	-0.5069382025
L__33pr			
L__a24pr			
L__p32pr	Area__p32__prime__L	0.7315258709	-0.675614868
L__a24			
L__d32	Area__dorsal__32__L	0.7691731506	
L__8BM	Area__8BM__L	0.690216105	-0.5914221799
L__p32			
L__10r	Area__10r__L	0.6981840449	-0.7333436803
L__47m			
L__8Av	Area__8Av__L	0.5085415152	-0.5754666817
L__8Ad	Area__8Ad__L		-0.7312351634
L__9m			
L__8BL	Area__8B__Lateral__L		-0.5938955577
L__9p			
L__10d	Area__10d__L	0.7438566253	-0.5159107131
L__8C	Area__8C__L	0.8609754685	-0.588660363
L__44	Area__44__L		-0.7973496142
L__45	Area__45__L	0.6617917694	-0.6845936807
L__47l	Area__47l__(47__lateral)__L	0.7223181174	-0.6191347531
L__a47r	Area__anterior__47r__L	0.6637874773	-0.577953874
L__6r	Rostral__Area__6__L		-0.9263914294
L__IFJa			
L__IFJp			
L__IFSp			
L__IFSa			
L__p9-46v			
L__46			
L__a9-46v			
L__9-46d			
L__9a			
L__10v	Area__10v__L	0.5765562363	-0.5900460132
L__a10p	Area__anterior__10p__L	0.6990769697	-0.6310089207
L__10pp	Polar__10p__L	0.7416709859	-0.6164405729
L__11l	Area__11l__L	0.7288441667	-0.6073071606
L__13l	Area__13l__L	0.6554894975	-0.5710149085
L__OFC	Orbital__Frontal__Complex__L	0.7163884928	-0.6057915255
L__47s	Area__47s__L	0.6998604156	-0.5920128651
L__LIPd			
L__6a	Area__6__anterior__L	0.6641752959	-0.6630818676
L__i6-8			
L__s6-8			
L__43			
L__OP4	Area__OP4-PV__L	0.7459153509	-0.6939638616
L__OP1	Area__OP1-SII__L	0.6667642743	-0.5495540258
L__OP2-3			
L__52			

Table S2 continued from previous page

Regions	Regionnames	Alertness	STAI
L__RI	RetroInsular__Cortex__L		-0.6263210457
L__PFcm	Area__PFcm__L		-0.8186291692
L__PoI2	Posterior__Insular__Area__2__L	0.7482736014	
L__TA2			
L__FOP4	Frontal__Opercular__Area__4__L	0.723702142	-0.5427195711
L__MI	Middle__Insular__Area__L		-0.6440284479
L__Pir	Pirform__Cortex__L	0.6118665432	-0.4186287005
L__AVI			
L__AAIC	Anterior__Agranular__Insula__Complex__L		-0.8006691445
L__FOP1	Frontal__Opercular__Area__1__L	0.784196708	-0.752867092
L__FOP3			
L__FOP2			
L__PFt			
L__AIP			
L__EC	Entorhinal__Cortex__L		-0.4284030451
L__PreS			
L__H	Hippocampus__L		-0.5222130539
L__ProS	ProStriate__Area__L	0.6921673762	-0.6196787525
L__PeEc	Perirhinal__Ectorhinal__Cortex__L	0.5928377557	-0.794700134
L__STGa	Area__STGa__L	0.6559863004	-0.6806141733
L__PBelt			
L__A5			
L__PHA1	ParaHippocampal__Area__1__L		-0.4859352194
L__PHA3	ParaHippocampal__Area__3__L	0.7479424996	-0.6453427967
L__STSda			
L__STSdp			
L__STSvp			
L__TGd	Area__TG__dorsal__L	0.6278459748	-0.5699713238
L__TE1a	Area__TE1__anterior__L		-0.7222251081
L__TE1p			
L__TE2a	Area__TE2__anterior__L	0.7506281218	-0.5794406939
L__TF	Area__TF__L	0.6758096816	-0.6062819428
L__TE2p	Area__TE2__posterior__L	0.7730521474	-0.6181219648
L__PHT			
L__PH			
L__TPOJ1			
L__TPOJ2			
L__TPOJ3			
L__DVT			
L__PGp			
L__IP2			
L__IP1	Area__IntraParietal__1__L	0.8575886992	-0.643397452
L__IP0			
L__PFop			
L__PF			
L__PFm	Area__PFm__Complex__L	0.6072435071	
L__PGi			
L__PGs			
L__V6A			
L__VMV1			
L__VMV3	VentroMedial__Visual__Area__3__L		-0.6124177853
L__PHA2			
L__V4t			
L__FST			
L__V3CD			
L__LO3			
L__VMV2			
L__31pd			
L__31a			
L__VVC	Ventral__Visual__Complex__L	0.6096625705	-0.641254079
L__25	Area__25__L	0.7683894941	-0.6239426792
L__s32			
L__pOFC	Posterior__OFC__Complex__L	0.6902042385	-0.476909234
L__PoI1	Area__Posterior__Insular__1__L	0.7437745658	
L__Ig	Insular__Granular__Complex__L		-0.7657404616
L__FOP5			
L__p10p			
L__p47r			
L__TGv	Area__TG__Ventral__L	0.6153989645	-0.7758510351
L__MBelt	Medial__Belt__Complex__L	0.7697931548	-0.8687409614
L__LBelt			
L__A4			
L__STSva			
L__TE1m			
L__PI			
L__a32pr			
L__p24			
R__V1	Primary__Visual__Cortex__L	0.6874433941	-0.5730697859
R__MST			
R__V6			
R__V2	Second__Visual__Area__L	0.5326059425	-0.5944010548
R__V3	Third__Visual__Area__L	0.7578349045	-0.4869629994
R__V4	Fourth__Visual__Area__L	0.716529107	-0.5094691718
R__V8			
R__4	Primary__Motor__Cortex__L	0.6382415323	-0.6618849775
R__3b	Primary__Sensory__Cortex__L	0.7650741633	
R__FEF	Frontal__Eye__Fields__L		-0.5984028469
R__PEF			
R__55b	Area__55b__L		-0.7112901836
R__V3A			
R__RSC	RetroSplenial__Complex__L	0.5907156605	-0.5947546157
R__POS2	Parieto-Occipital__Sulcus__Area__2__L		-0.6931131623
R__V7			
R__IPS1			
R__FFC	Fusiform__Face__Complex__L	0.6005742204	-0.6947222872
R__V3B			
R__RO1			
R__RO2			
R__PIT			
R__MT			
R__A1	Primary__Auditory__Cortex__L	0.8738314156	-0.7092114276
R__PSR			
R__SFR	R__SFR	0.6464375283	-0.6515970192
R__PCV			
R__STV			
R__7Pm			
R__7m			
R__POS1	Parieto-Occipital__Sulcus__Area__1__L		-0.5367632791

Table S2 continued from previous page

Regions	Regionnames	Alertness	STAI
R_23d	Area_23d_L	0.7112487783	-0.5843798111
R_v23ab	Area_ventral_23_a+b_L	0.7299172172	-0.7468606939
R_d23ab			
R_31pv			
R_5m	Area_5m_L	0.7709546963	-0.6664646604
R_5mv	Area_5m_ventral_L		-0.7745110574
R_23c	Area_23c_L	0.7200542848	-0.6857923962
R_5R	R_5R		-0.7228487218
R_24dd	Dorsal_Area_24d_L	0.6966820997	-0.5746949933
R_24dv	Ventral_Area_24d_L	0.7963316694	-0.6158676048
R_7AR			
R_SCEF	Supplementary_and_Cingulate_Eye_Field_L	0.6119785868	-0.6546937868
R_6ma	Area_6m_anterior_L		-0.6991809453
R_7Am	Medial_Area_7A_L	0.7692577788	-0.554429215
R_7PR			
R_7PC	Area_7PC_L	0.7316551012	-0.6304846477
R_RIPv			
R_VIP			
R_MIP			
R_1	Area_1_L	0.6071025831	-0.7094411648
R_2	Area_2_L	0.6929641234	-0.6691236585
R_3a	Area_3a_L	0.7359178385	-0.5977302137
R_6d			
R_6mp			
R_6v	Ventral_Area_6_L	0.8292991991	-0.6563858284
R_p24pr	Area_Posterior_24_prime_L	0.6971465364	-0.6327892845
R_33pr	Area_33_prime_L	0.692645819	-0.5931392131
R_a24pr	Anterior_24_prime_L		-0.7079320294
R_p32pr	Area_p32_prime_L	0.7571963124	-0.5288750021
R_a24			
R_d32	Area_dorsal_32_L	0.7200700897	-0.5800600554
R_8BM	Area_8BM_L	0.5926980829	-0.6176758007
R_p32			
R_10r			
R_47m			
R_8Av	Area_8Av_L	0.6567374329	-0.6520382755
R_8Ad	Area_8Ad_L		-0.8156491425
R_9m	Area_9_Middle_L	0.7981244401	
R_8BR	R_8BR		-0.4848303926
R_9p	Area_9_Posterior_L	0.7019749723	-0.718718189
R_10d	Area_10d_L	0.7369458519	-0.598410622
R_8C	Area_8C_L	0.6082063795	-0.5846514076
R_44	Area_44_L	0.5459571182	
R_45	Area_45_L	0.6707024386	-0.5544443266
R_47R			
R_a47r	Area_anterior_47r_L		-0.4905904426
R_6r	Rostral_Area_6_L	0.7697334516	-0.5527196287
R_IFJa			
R_IFJp			
R_IFSp			
R_IFSa			
R_p9-46v			
R_46	Area_46_L	0.7014628196	
R_a9-46v			
R_9-46d			
R_9a			
R_10v	Area_10v_L	0.7247205403	-0.6167074343
R_a10p			
R_10pp	Polar_10p_L	0.6867911704	-0.6504565278
R_11R	R_11R	0.7306627625	-0.5698144541
R_13R			
R_OFC	Orbital_Frontal_Complex_L	0.7321611691	-0.6024361512
R_47s	Area_47s_L	0.6623153409	-0.5978083698
R_RIPd			
R_6a			
R_i6-8			
R_s6-8			
R_43	Area_43_L	0.775197805	-0.4668445125
R_OP4	Area_OP4-PV_L	0.6879867399	-0.5816634047
R_OP1	Area_OP1-SII_L	0.8521975386	
R_OP2-3			
R_52			
R_RI			
R_PFcm	Area_PFcm_L	0.7453720938	-0.595512827
R_PoI2	Posterior_Insular_Area_2_L	0.7240557314	-0.5814447519
R_TA2	Area_TA2_L	0.8311546095	-0.6130964232
R_FOP4			
R_MI			
R_Pir	Pirform_Cortex_L	0.7457426987	-0.4848549514
R_AVI			
R_AAIC	Anterior_Agranular_Insula_Complex_L		-0.5088562341
R_FOP1	Frontal_Opercular_Area_1_L	0.6252983932	
R_FOP3			
R_FOP2			
R_PFt			
R_AIP			
R_EC			
R_PreS			
R_H			
R_ProS	ProStriate_Area_L		-0.8079074622
R_PeEc	Perirhinal_Ectorhinal_Cortex_L	0.5102326623	-0.6670102476
R_STGa	Area_STGa_L	0.7289104126	-0.7396721521
R_PBeRt			
R_A5			
R_PHA1	ParaHippocampal_Area_1_L		-0.5026512247
R_PHA3			
R_STSda			
R_STSdp			
R_STSvp			
R_TGd	Area_TG_dorsal_L		-0.4623196393
R_TE1a	Area_TE1_anterior_L		-0.4791342669
R_TE1p			
R_TE2a	Area_TE2_anterior_L	0.7046407436	-0.589247027
R_TF	Area_TF_L	0.5948267614	-0.638688079
R_TE2p	Area_TE2_posterior_L	0.7246782539	-0.555895272
R_PHT			
R_PH			
R_TPOJ1			

Table S2 continued from previous page

Regions	Regionnames	Alertness	STAI
R__TPOJ2			
R__TPOJ3			
R__DVT			
R__PGp			
R__IP2			
R__IP1			
R__IP0			
R__PFop			
R__PF			
R__PFm			
R__PGi			
R__PGs			
R__V6A			
R__VMV1	VentroMedial__Visual__Area__1__L		-0.5268687264
R__VMV3			
R__PHA2			
R__V4t			
R__FST			
R__V3CD			
R__RO3			
R__VMV2			
R__31pd			
R__31a	Area__31a__L	0.8796721381	
R__VVC	Ventral__Visual__Complex__L	0.5112225186	-0.6196265918
R__25	Area__25__L		-0.6347407581
R__s32			
R__pOFC	Posterior__OFC__Complex__L	0.6933430917	-0.4263105482
R__PoI1	Area__Posterior__Insular__1__L	0.7749977999	-0.499842001
R__Ig	Insular__Granular__Complex__L		-0.7991446499
R__FOP5			
R__p10p	Area__posterior__10p__L	0.6311187083	-0.527709087
R__p47r			
R__TGv	Area__TG__Ventral__L	0.5906502192	-0.6463799086
R__MBeRt	R__MBeRt	0.8298102805	-0.7055169551
R__RBeRt			
R__A4	Auditory__4__Complex__L	0.8758470793	-0.6225009734
R__STSva			
R__TE1m			
R__PI			
R__a32pr			
R__p24	Area__posterior__24__L		-0.7439768595
L__Cerebellum	L__Cerebellum		-0.4436726223
L__Thalamus			
L__Caudate			
L__Putamen			
L__Pallidum			
L__Hippocampus	L__Hippocampus		-0.5219331671
L__Amygdala	L__Amygdala	0.5915973195	-0.5497763933
L__Accumbens			
L__VentralDC	L__VentralDC		-0.4734314154
R__Cerebellum			
R__Thalamus			
R__Caudate			
R__Putamen			
R__Pallidum	R__Pallidum	0.4986542478	
R__Hippocampus	R__Hippocampus		-0.5589141477
R__Amygdala			
R__Accumbens			
R__VentralDC	R__VentralDC	0.5760753721	-0.4957036278

Table S3. Abbreviations of Glasser parcellation (https://neuroimaging-core-docs.readthedocs.io/en/latest/pages/atlases.html)

regionName	regionLongName	cortex	x-cog	y-cog	z-cog
V1_L	Primary_Visual_Cortex_L	Primary_Visual	100.491589	41.138901	71.63704
MST_L	Medial_Superior_Temporal_Area_L	MT+_Complex_and_Neighboring_Visual_Areas	132.416667	58.901786	82.059524
V6_L	Sixth_Visual_Area_L	Dorsal_Stream_Visual	104.543112	44.481665	103.916749
V2_L	Second_Visual_Area_L	Early_Visual	102.236656	44.064791	74.401125
V3_L	Third_Visual_Area_L	Early_Visual	107.926111	40.632159	76.961153
V4_L	Fourth_Visual_Area_L	Early_Visual	120.481621	41.344329	69.955702
V8_L	Eighth_Visual_Area_L	Ventral_Stream_Visual	123.013193	51.8927	56.759015
4_L	Primary_Motor_Cortex_L	Somatosensory_and_Motor	119.338359	105.485002	126.65561
3b_L	Primary_Sensory_Cortex_L	Somatosensory_and_Motor	129.664059	103.257279	123.682747
FEF_L	Frontal_Eye_Fields_L	Premotor	132.579336	118.837638	121.922509
PEF_L	Premotor_Eye_Field_L	Premotor	136.772664	125.582985	112.956764
55b_L	Area_55b_L	Premotor	138.865752	124.683174	122.399165
V3A_L	Area_V3A_L	Dorsal_Stream_Visual	104.528505	33.597072	99.335131
RSC_L	RetroSplenial_Complex_L	Posterior_Cingulate	96.918149	86.60261	88.986951
POS2_L	Parieto-Occipital_Sulcus_Area_2_L	Posterior_Cingulate	98.677433	53.233641	109.394715
V7_L	Seventh_Visual_Area_L	Dorsal_Stream_Visual	114.850993	39.443709	100.271523
IPS1_L	IntraParietal_Sulcus_Area_1_L	Dorsal_Stream_Visual	114.19184	51.537326	110.430556
FFC_L	Fusiform_Face_Complex_L	Ventral_Stream_Visual	133.940136	69.78536	52.618797
V3B_L	Area_V3B_L	Dorsal_Stream_Visual	117.755656	42.828054	88.434389
LO1_L	Area_Lateral_Occipital_1_L	MT+_Complex_and_Neighboring_Visual_Areas	132.592541	39.361878	78.374309
LO2_L	Area_Lateral_Occipital_2_L	MT+_Complex_and_Neighboring_Visual_Areas	137.691983	45.555907	68.914557
PIT_L	Posterior_InferoTemporal_complex_L	Ventral_Stream_Visual	137.394161	48.92805	61.153285
MT_L	Middle_Temporal_Area_L	MT+_Complex_and_Neighboring_Visual_Areas	133.489855	55.521739	82.907246
A1_L	Primary_Auditory_Cortex_L	Early_Auditory	135.998896	101.994481	82.398455
PSL_L	PeriSylvian_Language_Area_L	Temporo-Parieto_Occipital_Junction	150.183112	78.426117	97.249877
SFL_L	Superior_Frontal_Language_Area_L	Dorsolateral_Prefrontal	98.31097	142.730684	135.373291
PCV_L	PreCuneus_Visual_Area_L	Posterior_Cingulate	96.358639	76.151178	120.194372
STV_L	Superior_Temporal_Visual_Area_L	Temporo-Parieto-Occipital_Junction	152.198435	74.187814	89.932923
7Pm_L	Medial_Area_7P_L	Superior_Parietal	95.248227	58.274232	121.498818
7m_L	Area_7m_L	Posterior_Cingulate	95.552556	64.056289	105.57668
POS1_L	Parieto-Occipital_Sulcus_Area_1_L	Posterior_Cingulate	101.731298	67.452417	84.880916
23d_L	Area_23d_L	Posterior_Cingulate	93.867052	106.364162	109.955684
v23ab_L	Area_ventral_23_a+b_L	Posterior_Cingulate	94.704348	70.024638	91.217391
d23ab_L	Area_dorsal_23_a+b_L	Posterior_Cingulate	93.768848	84.202983	102.524441
31pv_L	Area_31p_ventral_L	Posterior_Cingulate	100.042751	81.552045	104.622677
5m_L	Area_5m_L	Paracentral_Lobular_and_Mid_Cingulate	96.894004	83.917559	134.828694
5mv_L	Area_5m_ventral_L	Paracentral_Lobular_and_Mid_Cingulate	104.643979	88.134031	121.951832
23c_L	Area_23c_L	Paracentral_Lobular_and_Mid_Cingulate	102.515493	96.142723	113.688263
5L_L	Area_5L_L	Paracentral_Lobular_and_Mid_Cingulate	105.121097	76.89642	144.418888
24dd_L	Dorsal_Area_24d_L	Paracentral_Lobular_and_Mid_Cingulate	96.658505	110.468299	121.557499
24dv_L	Ventral_Area_24d_L	Paracentral_Lobular_and_Mid_Cingulate	100.068306	126.534153	115.301913
7AL_L	Lateral_Area_7A_L	Superior_Parietal	112.239867	73.178073	137.489037
SCEF_L	Supplementary_and_Cingulate_Eye_Field_L	Paracentral_Lobular_and_Mid_Cingulate	96.643496	131.532274	128.125124
6ma_L	Area_6m_anterior_L	Paracentral_Lobular_and_Mid_Cingulate	110.592627	130.136617	139.421933
7Am_L	Medial_Area_7A_L	Superior_Parietal	97.517305	65.146285	132.871251
7Pl_L	Lateral_Area_7P_L	Superior_Parietal	105.278114	51.702539	127.938331
7PC_L	Area_7PC_L	Superior_Parietal	128.238394	78.294856	131.844417
LIPv_L	Area_Lateral_IntraParietal_ventral_L	Superior_Parietal	121.655546	69.556357	124.250676
VIP_L	Ventral_IntraParietal_Complex_L	Superior_Parietal	114.787443	60.903859	134.815566
MIP_L	Medial_IntraParietal_Area_L	Superior_Parietal	115.970641	58.793594	119.152135
1_L	Area_1_L	Somatosensory_and_Motor	138.150337	99.962157	127.34085
2_L	Area_2_L	Somatosensory_and_Motor	129.508165	92.68818	123.900078
3a_L	Area_3a_L	Somatosensory_and_Motor	125.373109	105	114.733613
6d_L	Dorsal_area_6_L	Premotor	124.128205	111.937022	138.11426
6mp_L	Area_6mp_L	Paracentral_Lobular_and_Mid_Cingulate	100.026417	110.678674	141.063401
6v_L	Ventral_Area_6_L	Premotor	149.799006	129.681108	103.925426
p24pr_L	Area_Posterior_24_prime_L	Anterior_Cingulate_and_Medial_Prefrontal	94.447546	124.308951	110.85948
33pr_L	Area_33_prime_L	Anterior_Cingulate_and_Medial_Prefrontal	94.083624	134.923345	100.006969
a24pr_L	Anterior_24_prime_L	Anterior_Cingulate_and_Medial_Prefrontal	95.249415	144.104215	101.338407
p32pr_L	Area_p32_prime_L	Anterior_Cingulate_and_Medial_Prefrontal	99.010823	140.837662	110.227273
a24_L	Area_a24_L	Anterior_Cingulate_and_Medial_Prefrontal	96.046431	166.310464	66.976438
d32_L	Area_dorsal_32_L	Anterior_Cingulate_and_Medial_Prefrontal	98.638734	168.099317	96.929236
8BM_L	Area_8BM_L	Anterior_Cingulate_and_Medial_Prefrontal	95.909177	158.968616	116.162149
p32_L	Area_p32_L	Anterior_Cingulate_and_Medial_Prefrontal	101.628302	174.318868	67.658491
10r_L	Area_10r_L	Anterior_Cingulate_and_Medial_Prefrontal	96.816092	176.014368	59.294061
47m_L	Area_47m_L	Orbital_and_Polar_Frontal	127.422402	156.510121	55.184885
8Av_L	Area_8Av_L	Dorsolateral_Prefrontal	128.190759	142.505809	124.802756
8Ad_L	Area_8Ad_L	Dorsolateral_Prefrontal	113.687799	154.661483	115.801037
9m_L	Area_9_Middle_L	Anterior_Cingulate_and_Medial_Prefrontal	97.019809	180.108831	93.67494
8BL_L	Area_8B_Lateral_L	Dorsolateral_Prefrontal	101.401615	163.951564	124.369828
9p_L	Area_9_Posterior_L	Dorsolateral_Prefrontal	109.307738	173.069108	109.713526
10d_L	Area_10d_L	Orbital_and_Polar_Frontal	100.894288	191.564911	78.3023
8C_L	Area_8C_L	Dorsolateral_Prefrontal	134.157612	139.145205	109.267606
44_L	Area_44_L	Inferior_Frontal	144.510015	141.238315	88.001027
45_L	Area_45_L	Inferior_Frontal	141.73524	152.514118	73.734873
47l_L	Area_47l_(47_lateral)_L	Inferior_Frontal	136.797521	154.852273	60.41374
a47r_L	Area_anterior_47r_L	Inferior_Frontal	131.095281	174.396953	59.360514
6r_L	Rostral_Area_6_L	Premotor	143.753559	132.158363	88.451957
IFJa_L	Area_IFJa_L	Inferior_Frontal	131.411067	137.512846	97.774704
IFJp_L	Area_IFJp_L	Inferior_Frontal	131.397321	127.672619	101.36756
IFSp_L	Area_IFSp_L	Inferior_Frontal	137.874185	149.780056	91.685927
IFSa_L	Area_IFSa_L	Inferior_Frontal	137.383825	159.179718	80.8819
p9-46v_L	Area_posterior_9-46v_L	Dorsolateral_Prefrontal	138.145009	155.665913	98.46629
46_L	Area_46_L	Dorsolateral_Prefrontal	127.033498	164.498689	105.07457
a9-46v_L	Area_anterior_9-46v_L	Dorsolateral_Prefrontal	131.147188	176.872535	80.33382
9-46d_L	Area_9-46d_L	Dorsolateral_Prefrontal	120.191583	172.262563	93.433103
9a_L	Area_9_anterior_L	Dorsolateral_Prefrontal	112.60695	183.845628	95.711763
10v_L	Area_10v_L	Anterior_Cingulate_and_Medial_Prefrontal	95.085146	179.625174	52.61453
a10p_L	Area_anterior_10p_L	Orbital_and_Polar_Frontal	116.405009	185.963731	63.137306
10pp_L	Polar_10p_L	Orbital_and_Polar_Frontal	103.361926	187.352144	54.227239
11l_L	Area_11l_L	Orbital_and_Polar_Frontal	115.577956	172.905411	55.238477
13l_L	Area_13l_L	Orbital_and_Polar_Frontal	113.105234	153.630846	50.639198
OFC_L	Orbital_Frontal_Complex_L	Orbital_and_Polar_Frontal	101.248092	155.905125	46.772628
47s_L	Area_47s_L	Orbital_and_Polar_Frontal	123.285457	146.215144	51.115986
LIPd_L	Area_Lateral_IntraParietal_dorsal_L	Superior_Parietal	120.72028	70.784965	112.674825
6a_L	Area_6_anterior_L	Premotor	115.421955	121.769876	126.648432
i6-8_L	Inferior_6-8_Transitional_Area_L	Dorsolateral_Prefrontal	119.66028	129.204864	127.283714
s6-8_L	Superior_6-8_Transitional_Area_L	Dorsolateral_Prefrontal	112.448914	151.434433	128.003218
43_L	Area_43_L	Posterior_Opercular	147.447146	125.598309	80.808668
OP4_L	Area_OP4-PV_L	Posterior_Opercular	151.038095	113.658333	86.099405
OP1_L	Area_OP1-SII_L	Posterior_Opercular	138.126662	103.983905	90.311407
OP2-3_L	Area_OP2-3-VS_L	Posterior_Opercular	131.5	109.658263	89.165266
52_L	Area_52_L	Early_Auditory	130.277162	105.017738	70.645233
RI_L	RetroInsular_Cortex_L	Early_Auditory	130.666225	90.109934	91.313907
PFcm_L	Area_PFcm_L	Early_Auditory	141.793228	93.845101	92.239193
PoI2_L	Posterior_Insular_Area_2_L	Insular_and_Frontal_Opercular	131.140744	121.382156	71.041031

Table S3 continued from previous page

regionName	regionLongName	cortex	x-cog	y-cog	z-cog
TA2_L	Area_TA2_L	Auditory_Association	141.08604	129.967515	63.526778
FOP4_L	Frontal_Opercular_Area_4_L	Insular_and_Frontal_Opercular	133.429119	138.892173	76.685824
MI_L	Middle_Insular_Area_L	Insular_and_Frontal_Opercular	127.923709	136.443075	73.143779
Pir_L	Pirform_Cortex_L	Insular_and_Frontal_Opercular	122.938706	130.870602	53.682179
AVI_L	Anterior_Ventral_Insular_Area_L	Insular_and_Frontal_Opercular	121.113485	150.508678	67.823765
AAIC_L	Anterior_Agranular_Insula_Complex_L	Insular_and_Frontal_Opercular	126.225504	139.71902	60.595101
FOP1_L	Frontal_Opercular_Area_1_L	Posterior_Opercular	140.305709	128.198895	75.653775
FOP3_L	Frontal_Opercular_Area_3_L	Insular_and_Frontal_Opercular	126	128.96978	84.425824
FOP2_L	Frontal_Opercular_Area_2_L	Insular_and_Frontal_Opercular	133.704403	121.251572	85.752621
PFt_L	Area_PFt_L	Inferior_Parietal	146.277662	100.807933	106.926931
AIP_L	Anterior_IntraParietal_Area_L	Superior_Parietal	130.372549	87.01364	112.687127
EC_L	Entorhinal_Cortex_L	Medial_Temporal	111.463631	115.664695	42.698403
PreS_L	PreSubiculum_L	Medial_Temporal	109.856877	94.634758	60.597584
H_L	Hippocampus_L	Medial_Temporal	120.203438	102.589542	54.63467
ProS_L	ProStriate_Area_L	Posterior_Cingulate	112.538922	71.057884	75.091816
PeEc_L	Perirhinal_Ectorhinal_Cortex_L	Medial_Temporal	120.292051	116.757071	38.222388
STGa_L	Area_STGa_L	Auditory_Association	139.957301	140.979968	50.18292
PBelt_L	ParaBelt_Complex_L	Early_Auditory	144.62098	100.147014	79.898162
A5_L	Auditory_5_Complex_L	Auditory_Association	152.259123	112.858549	68.432144
PHA1_L	ParaHippocampal_Area_1_L	Medial_Temporal	111.705833	90.678333	55.309167
PHA3_L	ParaHippocampal_Area_3_L	Medial_Temporal	124.479062	90.840871	51.123953
STSda_L	Area_STSd_anterior_L	Auditory_Association	143.831461	123.554073	57.391854
STSdp_L	Area_STSd_posterior_L	Auditory_Association	143.062832	94.156637	72.083186
STSvp_L	Area_STSv_posterior_L	Auditory_Association	146.913625	91.085158	67.189173
TGd_L	Area_TG_dorsal_L	Lateral_Temporal	127.26011	137.751977	35.261304
TE1a_L	Area_TE1_anterior_L	Lateral_Temporal	150.202228	122.362624	47.172277
TE1p_L	Area_TE1_posterior_L	Lateral_Temporal	152.940325	78.805713	61.515946
TE2a_L	Area_TE2_anterior_L	Lateral_Temporal	148.079856	104.370685	42.029109
TF_L	Area_TF_L	Medial_Temporal	132.406298	105.142089	41.219662
TE2p_L	Area_TE2_posterior_L	Lateral_Temporal	142.182155	83.665679	49.921881
PHT_L	Area_PHT_L	Lateral_Temporal	150.083714	66.180795	74.12733
PH_L	Area_PH_L	MT+_Complex_and_Neighboring_Visual_Areas	139.578644	61.953921	65.579087
TPOJ1_L	Area_TemporoParietoOccipital_Junction_1_L	Temporo-Parieto-Occipital_Junction	144.16796	79.669019	81.137615
TPOJ2_L	Area_TemporoParietoOccipital_Junction_2_L	Temporo-Parieto-Occipital_Junction	143.28	62.095686	87.299608
TPOJ3_L	Area_TemporoParietoOccipital_Junction_3_L	Temporo-Parieto-Occipital_Junction	133.675835	54.257367	91.510806
DVT_L	Dorsal_Transitional_Visual_Area_L	Posterior_Cingulate	108.042324	53.086307	103.566805
PGp_L	Area_PGp_L	Inferior_Parietal	129.425332	40.278761	94.142146
IP2_L	Area_IntraParietal_2_L	Inferior_Parietal	131.750374	77.285501	114.758595
IP1_L	Area_IntraParietal_1_L	Inferior_Parietal	121.635182	55.480069	111.34922
IP0_L	Area_IntraParietal_0_L	Inferior_Parietal	122.033257	45.806193	97.033257
PFop_L	Area_PF_Opercular_L	Inferior_Parietal	154.842679	102.913551	96.221963
PF_L	Area_PF_Complex_L	Inferior_Parietal	151.894297	89.867502	108.288348
PFm_L	Area_PFm_Complex_L	Inferior_Parietal	140.702334	70.065862	118.414359
PGi_L	Area_PGi_L	Inferior_Parietal	138.62727	61.299798	99.072355
PGs_L	Area_PGs_L	Inferior_Parietal	131.808463	48.857461	110.436526
V6A_L	Area_V6A_L	Dorsal_Stream_Visual	112.534483	39.66092	114.188218
VMV1_L	VentroMedial_Visual_Area_1_L	Ventral_Stream_Visual	110.244105	74.319001	64.307906
VMV3_L	VentroMedial_Visual_Area_3_L	Ventral_Stream_Visual	119.618762	64.508982	61.017964
PHA2_L	ParaHippocampal_Area_2_L	Medial_Temporal	121.389831	90.169492	58.122881
V4t_L	Area_V4t_L	MT+_Complex_and_Neighboring_Visual_Areas	138.695811	48.185792	74.724954
FST_L	Area_FST_L	MT+_Complex_and_Neighboring_Visual_Areas	137.648765	57.787325	76.535983
V3CD_L	Area_V3CD_L	MT+_Complex_and_Neighboring_Visual_Areas	125.152542	36.195686	81.429892
LO3_L	Area_Lateral_Occipital_3_L	MT+_Complex_and_Neighboring_Visual_Areas	136.842415	48.964654	85.864507
VMV2_L	VentroMedial_Visual_Area_2_L	Ventral_Stream_Visual	119.360502	74.39185	66.366771
31pd_L	Area_31pd_L	Posterior_Cingulate	102.136519	73.851536	106.767918
31a_L	Area_31a_L	Posterior_Cingulate	95.984389	88.707989	115.960514
VVC_L	Ventral_Visual_Complex_L	Ventral_Stream_Visual	121.913003	74.920407	53.816752
25_L	Area_25_L	Anterior_Cingulate_and_Medial_Prefrontal	95.291898	147.049945	57.497225
s32_L	Area_s32_L	Anterior_Cingulate_and_Medial_Prefrontal	97.364516	159.174194	54.480645
pOFC_L	Posterior_OFC_Complex_L	Anterior_Cingulate_and_Medial_Prefrontal	104.455746	137.165786	52.015852
PoI1_L	Area_Posterior_Insular_1_L	Insular_and_Frontal_Opercular	129.839378	113.685665	68.664076
Ig_L	Insular_Granular_Complex_L	Insular_and_Frontal_Opercular	126.25974	110.064935	86.446429
FOP5_L	Area_Frontal_Opercular_5_L	Insular_and_Frontal_Opercular	125.928129	152.826518	75.615861
p10p_L	Area_posterior_10p_L	Orbital_and_Polar_Frontal	114.061006	189.546541	72.976101
p47r_L	Area_posterior_47r_L	Inferior_Frontal	134.951128	169.179699	72.101504
TGv_L	Area_TG_Ventral_L	Lateral_Temporal	127.497751	123.922939	25.901949
MBelt_L	Medial_Belt_Complex_L	Early_Auditory	136.939267	110.757068	73.956021
LBelt_L	Lateral_Belt_Complex_L	Early_Auditory	136.829565	99.368696	79.617391
A4_L	Auditory_4_Complex_L	Auditory_Association	155.165477	104.292219	78.188243
STSva_L	Area_STSv_anterior_L	Auditory_Association	142.689243	118.134462	52.832669
TE1m_L	Area_TE1_Middle_L	Lateral_Temporal	155.554922	101.395287	54.901178
PI_L	Para-Insular_Area_L	Insular_and_Frontal_Opercular	134.650549	124.293407	56.102198
a32pr_L	Area_anterior_32_prime_L	Anterior_Cingulate_and_Medial_Prefrontal	97.782313	155.883735	101.53927
p24_L	Area_posterior_24_L	Anterior_Cingulate_and_Medial_Prefrontal	95.442887	162.526279	84.888577
V1_R	Primary_Visual_Cortex_R	Primary_Visual	78.060375	44.539286	74.333474
MST_R	Medial_Superior_Temporal_Area_R	MT+_Complex_and_Neighboring_Visual_Areas	43.620295	63.751227	78.013093
V6_R	Sixth_Visual_Area_R	Dorsal_Stream_Visual	72.226868	49.256228	102.929715
V2_R	Second_Visual_Area_R	Early_Visual	77.026083	47.558042	76.305265
V3_R	Third_Visual_Area_R	Early_Visual	71.976821	41.580308	78.859282
V4_R	Fourth_Visual_Area_R	Early_Visual	57.656017	41.782393	71.003195
V8_R	Eighth_Visual_Area_R	Ventral_Stream_Visual	60.698264	51.949266	59.301736
4_R	Primary_Motor_Cortex_R	Somatosensory_and_Motor	60.980681	108.738321	126.822972
3b_R	Primary_Sensory_Cortex_R	Somatosensory_and_Motor	52.032115	105.85919	122.884387
FEF_R	Frontal_Eye_Fields_R	Premotor	46.370874	123.157282	123.201942
PEF_R	Premotor_Eye_Field_R	Premotor	45.069136	127.783951	109.748148
55b_R	Area_55b_R	Premotor	41.45439	127.938619	119.895993
V3A_R	Area_V3A_R	Dorsal_Stream_Visual	75.553398	36.584466	102.458252
RSC_R	RetroSplenial_Complex_R	Posterior_Cingulate	84.934198	92.434662	93.496756
POS2_R	Parieto-Occipital_Sulcus_Area_2_R	Posterior_Cingulate	80.662438	56.100589	108.736747
V7_R	Seventh_Visual_Area_R	Dorsal_Stream_Visual	62.285714	42.400534	103.668892
IPS1_R	IntraParietal_Sulcus_Area_1_R	Dorsal_Stream_Visual	64.710065	55.195753	111.066482
FFC_R	Fusiform_Face_Complex_R	Ventral_Stream_Visual	49.72947	75.126159	51.324834
V3B_R	Area_V3B_R	Dorsal_Stream_Visual	60.919386	48.422265	91.56238
LO1_R	Area_Lateral_Occipital_1_R	MT+_Complex_and_Neighboring_Visual_Areas	47.281977	45.175872	76.959302
LO2_R	Area_Lateral_Occipital_2_R	MT+_Complex_and_Neighboring_Visual_Areas	44.07	44.375	67.918333
PIT_R	Posterior_InferoTemporal_complex_R	Ventral_Stream_Visual	44.641614	49.954905	58.057753
MT_R	Middle_Temporal_Area_R	MT+_Complex_and_Neighboring_Visual_Areas	38.58547	57.830484	80.888889
A1_R	Primary_Auditory_Cortex_R	Early_Auditory	46.328479	105.454693	81.894822
PSL_R	PeriSylvian_Language_Area_R	Temporo-Parieto-Occipital_Junction	25.980366	89.316377	98.788934
SFL_R	Superior_Frontal_Language_Area_R	Dorsolateral_Prefrontal	82.702811	141.914659	137.409639
PCV_R	PreCuneus_Visual_Area_R	Posterior_Cingulate	84.876497	74.045369	121.958412
STV_R	Superior_Temporal_Visual_Area_R	Temporo-Parieto-Occipital_Junction	30.525124	81.598012	91.518498
7Pm_R	Medial_Area_7P_R	Superior_Parietal	84.893683	59.192604	121.912173
7m_R	Area_7m_R	Posterior_Cingulate	85.454762	66.283333	105.271429
POS1_R	Parieto-Occipital_Sulcus_Area_1_R	Posterior_Cingulate	79.376277	69.248778	87.420702
23d_R	Area_23d_R	Posterior_Cingulate	86.894973	103.731598	111.127469
v23ab_R	Area_ventral_23_a+b_R	Posterior_Cingulate	86.05698	74.521368	90.964387
d23ab_R	Area_dorsal_23_a+b_R	Posterior_Cingulate	86.84742	84.848518	104.53787

Table S3 continued from previous page

regionName	regionLongName	cortex	x-cog	y-cog	z-cog
31pv_R	Area_31p_ventral_R	Posterior_Cingulate	80.754159	82.767098	105.438078
5m_R	Area_5m_R	Paracentral_Lobular_and_Mid_Cingulate	85.168053	86.77371	136.587354
5mv_R	Area_5m_ventral_R	Paracentral_Lobular_and_Mid_Cingulate	79.398401	84.221852	127.565623
23c_R	Area_23c_R	Paracentral_Lobular_and_Mid_Cingulate	80.814159	89.430678	115.231268
5L_R	Area_5L_R	Paracentral_Lobular_and_Mid_Cingulate	77.196914	77.524614	145.837619
24dd_R	Dorsal_Area_24d_R	Paracentral_Lobular_and_Mid_Cingulate	84.408074	109.82229	124.363317
24dv_R	Ventral_Area_24d_R	Paracentral_Lobular_and_Mid_Cingulate	82.241719	124.339302	118.569382
7AL_R	Lateral_Area_7A_R	Superior_Parietal	66.808871	72.715323	139.629032
SCEF_R	Supplementary_and_Cingulate_Eye_Field_R	Paracentral_Lobular_and_Mid_Cingulate	84.455241	131.183536	131.043635
6ma_R	Area_6m_anterior_R	Paracentral_Lobular_and_Mid_Cingulate	69.698918	131.674072	137.728442
7Am_R	Medial_Area_7A_R	Superior_Parietal	81.616644	64.449523	132.899045
7Pl_R	Lateral_Area_7P_R	Superior_Parietal	78.082592	52.547649	129.927573
7PC_R	Area_7PC_R	Superior_Parietal	54.30504	78.639699	133.54244
LIPv_R	Area_Lateral_IntraParietal_ventral_R	Superior_Parietal	62.004614	69.61707	125.258362
VIP_R	Ventral_IntraParietal_Complex_R	Superior_Parietal	68.916533	63.23114	135.889246
MIP_R	Medial_IntraParietal_Area_R	Superior_Parietal	63.267232	58.20226	123.986441
1_R	Area_1_R	Somatosensory_and_Motor	41.860496	104.311709	125.768987
2_R	Area_2_R	Somatosensory_and_Motor	52.428189	95.303667	122.502292
3a_R	Area_3a_R	Somatosensory_and_Motor	58.537415	105.608844	118.408163
6d_R	Dorsal_area_6_R	Premotor	53.27913	114.122216	136.10668
6mp_R	Area_6mp_R	Paracentral_Lobular_and_Mid_Cingulate	75.523118	112.953763	140.165054
6v_R	Ventral_Area_6_R	Premotor	32.423866	132.818138	103.072554
p24pr_R	Area_Posterior_24_prime_R	Anterior_Cingulate_and_Medial_Prefrontal	85.71482	122.438623	112.543413
33pr_R	Area_33_prime_R	Anterior_Cingulate_and_Medial_Prefrontal	87.197935	136.562823	100.375215
a24pr_R	Anterior_24_prime_R	Anterior_Cingulate_and_Medial_Prefrontal	85.926439	145.473348	103.304904
p32pr_R	Area_p32_prime_R	Anterior_Cingulate_and_Medial_Prefrontal	82.475465	140.625212	112.576142
a24_R	Area_a24_R	Anterior_Cingulate_and_Medial_Prefrontal	85.878669	164.201566	70.600783
d32_R	Area_dorsal_32_R	Anterior_Cingulate_and_Medial_Prefrontal	83.510986	164.846197	98.791549
8BM_R	Area_8BM_R	Anterior_Cingulate_and_Medial_Prefrontal	85.561807	157.006339	117.958003
p32_R	Area_p32_R	Anterior_Cingulate_and_Medial_Prefrontal	82.359393	174.618436	69.042007
10r_R	Area_10r_R	Anterior_Cingulate_and_Medial_Prefrontal	85.146262	172.751896	60.15493
47m_R	Area_47m_R	Orbital_and_Polar_Frontal	57.754386	157.371517	53.686275
8Av_R	Area_8Av_R	Dorsolateral_Prefrontal	51.352616	145.949195	120.685614
8Ad_R	Area_8Ad_R	Dorsolateral_Prefrontal	67.233301	158.5722	113.623772
9m_R	Area_9_Middle_R	Anterior_Cingulate_and_Medial_Prefrontal	83.868691	181.249145	91.301967
8BL_R	Area_8B_Lateral_R	Dorsolateral_Prefrontal	78.91626	168.845999	120.171207
9p_R	Area_9_Posterior_R	Dorsolateral_Prefrontal	71.348837	176.911205	105.233263
10d_R	Area_10d_R	Orbital_and_Polar_Frontal	81.086781	193.730216	74.665468
8C_R	Area_8C_R	Dorsolateral_Prefrontal	50.37308	145.14594	108.576811
44_R	Area_44_R	Inferior_Frontal	37.055203	145.308467	85.935472
45_R	Area_45_R	Inferior_Frontal	39.515005	153.265247	72.484511
47l_R	Area_47l_(47_lateral)_R	Inferior_Frontal	46.146087	158.131594	56.831304
a47r_R	Area_anterior_47r_R	Inferior_Frontal	52.625557	178.539747	62.249629
6r_R	Rostral_Area_6_R	Premotor	39.694803	135.156311	86.720594
IFJa_R	Area_IFJa_R	Inferior_Frontal	50.829828	143.133843	95.51434
IFJp_R	Area_IFJp_R	Inferior_Frontal	54.052897	133.420655	99.677582
IFSp_R	Area_IFSp_R	Inferior_Frontal	44.515479	155.734341	89.435565
IFSa_R	Area_IFSa_R	Inferior_Frontal	42.045036	164.740532	73.742068
p9-46v_R	Area_posterior_9-46v_R	Dorsolateral_Prefrontal	43.648143	159.269405	97.278164
46_R	Area_46_R	Dorsolateral_Prefrontal	54.973373	167.290598	102.286325
a9-46v_R	Area_anterior_9-46v_R	Dorsolateral_Prefrontal	50.407407	178.769841	79.984127
9-46d_R	Area_9-46d_R	Dorsolateral_Prefrontal	61.307805	176.773458	93.881321
9a_R	Area_9_anterior_R	Dorsolateral_Prefrontal	70.836969	188.89409	90.121777
10v_R	Area_10v_R	Anterior_Cingulate_and_Medial_Prefrontal	87.511155	176.704016	52.646009
a10p_R	Area_anterior_10p_R	Orbital_and_Polar_Frontal	66.79639	188.730686	61.363177
10pp_R	Polar_10p_R	Orbital_and_Polar_Frontal	79.325648	188.469741	53.417291
11l_R	Area_11l_R	Orbital_and_Polar_Frontal	65.791587	172.746973	54.321224
13l_R	Area_13l_R	Orbital_and_Polar_Frontal	70.701739	152.646957	50.322609
OFC_R	Orbital_Frontal_Complex_R	Orbital_and_Polar_Frontal	83.615202	158.638955	45.933017
47s_R	Area_47s_R	Orbital_and_Polar_Frontal	60.445039	146.166964	51.135472
LIPd_R	Area_Lateral_IntraParietal_dorsal_R	Superior_Parietal	60.154362	70.736018	115.630872
6a_R	Area_6_anterior_R	Premotor	64.183119	124.292844	124.65945
i6-8_R	Inferior_6-8_Transitional_Area_R	Dorsolateral_Prefrontal	58.342047	134.887653	128.925473
s6-8_R	Superior_6-8_Transitional_Area_R	Dorsolateral_Prefrontal	70.548089	149.926634	130.08693
43_R	Area_43_R	Posterior_Opercular	34.231572	127.220516	81.591523
OP4_R	Area_OP4-PV_R	Posterior_Opercular	31.065974	115.43372	85.110568
OP1_R	Area_OP1-SII_R	Posterior_Opercular	45.814286	107.14898	89.419388
OP2-3_R	Area_OP2-3-VS_R	Posterior_Opercular	51.053528	111.892944	90.335766
52_R	Area_52_R	Early_Auditory	52.031915	105.12766	73.634752
RI_R	RetroInsular_Cortex_R	Early_Auditory	46.93904	94.059663	90.975357
PFcm_R	Area_PFcm_R	Early_Auditory	42.446673	98.010939	94.233364
PoI2_R	Posterior_Insular_Area_2_R	Insular_and_Frontal_Opercular	50.080778	122.827214	71.63067
TA2_R	Area_TA2_R	Auditory_Association	39.836312	130.301644	63.779843
FOP4_R	Frontal_Opercular_Area_4_R	Insular_and_Frontal_Opercular	50.523336	141.361132	77.437643
MI_R	Middle_Insular_Area_R	Insular_and_Frontal_Opercular	52.77037	135.922222	73.7
Pir_R	Pirform_Cortex_R	Insular_and_Frontal_Opercular	57.555081	133.362084	52.703672
AVI_R	Anterior_Ventral_Insular_Area_R	Insular_and_Frontal_Opercular	58.445161	150.95914	67.72043
AAIC_R	Anterior_Agranular_Insula_Complex_R	Insular_and_Frontal_Opercular	54.936552	141.408966	60.337241
FOP1_R	Frontal_Opercular_Area_1_R	Posterior_Opercular	43.638509	131.004969	75.765217
FOP3_R	Frontal_Opercular_Area_3_R	Insular_and_Frontal_Opercular	56.149813	133.580524	83.445693
FOP2_R	Frontal_Opercular_Area_2_R	Insular_and_Frontal_Opercular	49.524038	122.612981	86.716346
PFt_R	Area_PFt_R	Inferior_Parietal	36.126206	103.276912	110.400148
AIP_R	Anterior_IntraParietal_Area_R	Superior_Parietal	53.147097	85.464516	115.539355
EC_R	Entorhinal_Cortex_R	Medial_Temporal	70.108232	115.451982	45.18064
PreS_R	PreSubiculum_R	Medial_Temporal	70.221751	95.334746	62.004237
H_R	Hippocampus_R	Medial_Temporal	60.712375	103.927258	54.33194
ProS_R	ProStriate_Area_R	Posterior_Cingulate	70.101423	77.854093	73.617438
PeEc_R	Perirhinal_Ectorhinal_Cortex_R	Medial_Temporal	63.003326	117.21583	36.977054
STGa_R	Area_STGa_R	Auditory_Association	41.594035	142.606208	49.973828
PBelt_R	ParaBelt_Complex_R	Early_Auditory	33.096591	107.575487	79.568994
A5_R	Auditory_5_Complex_R	Auditory_Association	29.134363	113.095062	68.029224
PHA1_R	ParaHippocampal_Area_1_R	Medial_Temporal	70.162458	92.5	55.005892
PHA3_R	ParaHippocampal_Area_3_R	Medial_Temporal	56.793319	89.695198	55.185804
STSda_R	Area_STSd_anterior_R	Auditory_Association	38.705061	125.180919	55.141361
STSdp_R	Area_STSd_posterior_R	Auditory_Association	41.526702	98.094126	69.690254
STSvp_R	Area_STSv_posterior_R	Auditory_Association	30.95466	98.034257	65.971788
TGd_R	Area_TG_dorsal_R	Lateral_Temporal	55.264395	140.071941	34.559763
TE1a_R	Area_TE1_anterior_R	Lateral_Temporal	32.156938	125.713112	43.546788
TE1p_R	Area_TE1_posterior_R	Lateral_Temporal	27.790845	84.652057	59.44899
TE2a_R	Area_TE2_anterior_R	Lateral_Temporal	35.763051	107.785779	39.880513
TF_R	Area_TF_R	Medial_Temporal	49.626318	105.570338	41.435842
TE2p_R	Area_TE2_posterior_R	Lateral_Temporal	41.843798	89.348137	49.277693
PHT_R	Area_PHT_R	Lateral_Temporal	30.004594	73.093109	65.673201
PH_R	Area_PH_R	MT+_Complex_and_Neighboring_Visual_Areas	41.367731	64.734587	61.13861
TPOJ1_R	Area_TemporoParietoOccipital_Junction_1_R	Temporo-Parieto-Occipital_Junction	36.339798	82.305051	82.842828
TPOJ2_R	Area_TemporoParietoOccipital_Junction_2_R	Temporo-Parieto-Occipital_Junction	34.431659	69.409508	81.67477
TPOJ3_R	Area_TemporoParietoOccipital_Junction_3_R	Temporo-Parieto-Occipital_Junction	44.569097	65.107726	91.621328
DVT_R	Dorsal_Transitional_Visual_Area_R	Posterior_Cingulate	71.658746	57.291089	104.820462

Table S3 continued from previous page

regionName	regionLongName	cortex	x-cog	y-cog	z-cog
PGp_R	Area_PGp_R	Inferior_Parietal	45.491584	48.937135	98.336654
IP2_R	Area_IntraParietal_2_R	Inferior_Parietal	49.719313	79.054936	117.186266
IP1_R	Area_IntraParietal_1_R	Inferior_Parietal	56.10469	57.245394	114.665829
IP0_R	Area_IntraParietal_0_R	Inferior_Parietal	56.220528	48.778455	98.620935
PFop_R	Area_PF_Opercular_R	Inferior_Parietal	28.000702	105.724719	95.294944
PF_R	Area_PF_Complex_R	Inferior_Parietal	29.656612	96.343693	111.425046
PFm_R	Area_PFm_Complex_R	Inferior_Parietal	38.024068	76.292314	117.520769
PGi_R	Area_PGi_R	Inferior_Parietal	37.886767	66.21809	101.865327
PGs_R	Area_PGs_R	Inferior_Parietal	45.735542	57.616193	114.445321
V6A_R	Area_V6A_R	Dorsal_Stream_Visual	66.971053	43.206579	117.961842
VMV1_R	VentroMedial_Visual_Area_1_R	Ventral_Stream_Visual	71.942782	73.198944	63.584507
VMV3_R	VentroMedial_Visual_Area_3_R	Ventral_Stream_Visual	62.83642	64.92284	61.896605
PHA2_R	ParaHippocampal_Area_2_R	Medial_Temporal	60.39823	91.648968	57.716814
V4t_R	Area_V4t_R	MT+_Complex_and_Neighboring_Visual_Areas	41.209816	53.52638	69.720245
FST_R	Area_FST_R	MT+_Complex_and_Neighboring_Visual_Areas	38.91249	62.962609	72.511535
V3CD_R	Area_V3CD_R	MT+_Complex_and_Neighboring_Visual_Areas	51.141138	43.050328	83.778993
LO3_R	Area_Lateral_Occipital_3_R	MT+_Complex_and_Neighboring_Visual_Areas	41.930187	53.481809	85.433628
VMV2_R	VentroMedial_Visual_Area_2_R	Ventral_Stream_Visual	62.482	72.616	65.532
31pd_R	Area_31pd_R	Posterior_Cingulate	77.37785	74.687296	107.338762
31a_R	Area_31a_R	Posterior_Cingulate	83.9	85.809091	114.561039
VVC_R	Ventral_Visual_Complex_R	Ventral_Stream_Visual	61.276977	79.81999	52.749876
25_R	Area_25_R	Anterior_Cingulate_and_Medial_Prefrontal	87.037037	143.861454	58.698217
s32_R	Area_s32_R	Anterior_Cingulate_and_Medial_Prefrontal	86.948307	156.390374	55.54902
pOFC_R	posterior_OFC_Complex_R	Anterior_Cingulate_and_Medial_Prefrontal	77.656757	138.762838	52.856757
PoI1_R	Area_Posterior_Insular_1_R	Insular_and_Frontal_Opercular	52.45642	115.341143	68.178069
Ig_R	Insular_Granular_Complex_R	Insular_and_Frontal_Opercular	54.984127	112.0839	86.482993
FOP5_R	Area_Frontal_Opercular_5_R	Insular_and_Frontal_Opercular	53.738178	153.878893	75.189158
p10p_R	Area_posterior_10p_R	Orbital_and_Polar_Frontal	66.879454	187.415677	72.800475
p47r_R	Area_posterior_47r_R	Inferior_Frontal	44.467343	172.381737	65.509195
TGv_R	Area_TG_Ventral_R	Lateral_Temporal	56.653745	123.570388	25.638589
MBelt_R	Medial_Belt_Complex_R	Early_Auditory	43.806818	111.762784	74.931818
LBelt_R	Lateral_Belt_Complex_R	Early_Auditory	41.808564	101.870277	81.278338
A4_R	Auditory_4_Complex_R	Auditory_Association	25.519804	111.76766	76.545529
STSva_R	Area_STSv_anterior_R	Auditory_Association	32.924419	117.527907	54.337791
TE1m_R	Area_TE1_Middle_R	Lateral_Temporal	26.146605	102.236497	53.246528
PI_R	Para-Insular_Area_R	Insular_and_Frontal_Opercular	47.013363	123.157016	57.926503
a32pr_R	Area_anterior_32_prime_R	Anterior_Cingulate_and_Medial_Prefrontal	81.723096	153.990326	102.516324
p24_R	Area_posterior_24_R	Anterior_Cingulate_and_Medial_Prefrontal	86.096222	161.202423	88.541696

Table S4. Characteristics of 23 participants included in the final analysis

Age	24.43 (SD 3.9)
Heigh	180.81 (SD 6.74)
weight	81.17 (SD 9.48)
BMI	25.00 (SD 2.68)
Education	16 High school diploma (Abitur)
	1 University Diploma
	3 Master
	3 Batchelor
Smoking	4 yes
	19 no
Substances tried	10 yes
	13 no

References

1. M Sasaki, et al., Neuromelanin magnetic resonance imaging of locus ceruleus and substantia nigra in Parkinson's disease. *Neuroreport* **17**, 1215–8 (2006).
2. M Barth, BA Poser, Advances in high-field bold fmri. *Materials* **4**, 1941–1955 (2011).
3. T Liebe, et al., Ketamine influences the locus coeruleus norepinephrine network, with a dependency on norepinephrine transporter genotype - a placebo controlled fMRI study. *Neuroimage Clin* **20**, 715–723 (2018).
4. T Liebe, et al., In vivo anatomical mapping of human locus coeruleus functional connectivity at 3 T MRI. *Hum Brain Mapp* **41**, 2136–2151 (2020).
5. JS Allen, et al., Effects of spatial transformation on regional brain volume estimates. *Neuroimage* **42**, 535–547 (2008).
6. MD Sacchet, B Knutson, Spatial smoothing systematically biases the localization of reward-related brain activity. *Neuroimage* **66**, 270–277 (2013).
